# Sea stars of the genus *Henricia* Gray, 1840 (Echinodermata, Asteroidea) from Vostok Bay, Sea of Japan

**DOI:** 10.7717/peerj.6585

**Published:** 2019-03-21

**Authors:** Anton Chichvarkhin, Olga Chichvarkhina, Daiki Wakita

**Affiliations:** 1 National Scientific Center of Marine Biology, Far East Branch of Russian Academy of Sciences, Vladivostok, Russia; 2 Far Eastern Federal University, Vladivostok, Russia; 3 Hokkaido University, Sapporo, Japan

**Keywords:** Echinasteridae, Local fauna, Western Pacific

## Abstract

We report seven species of the genus *Henricia* Gray, 1840 that were found in Vostok Bay, and two species from adjacent area, known from museum collection or seen in underwater footage. while existing literature reported no confirmed species from this area. Most of these species: *H. djakonovi, H. alexeyi, H. densispina, H. hayashii, H. granulifera, H. pacifica, H. asiatica*, and *H. oculata robusta* were reported from the Sea of Japan previously. *H. nipponica*, known from Japan, is reported from Russian seas for the first time. All studied taxa are re-described here using a range of morphological characters and partial 16S rRNA nucleotide sequences, life colorations of several species are reported for the first time, and an identification key is provided. Lectotype designations are fixed for studied series of species described by AM Djakonov.

## Introduction

Sea stars of the genus *Henricia* Gray, 1840, belonging to the family Echinasteridae (Asteroidea, Spinulosida), have been a challenge for systematists to sort out because of their high species diversity, similar appearance, confusing taxonomic history, and often broad geographic distribution, especially in the northern Pacific (Verrill, 1909; Verrill, 1914; Fisher, 1911; Fisher, 1928; Fisher, 1930; Hayashi, 1940; Shin & Rho, 1996; Djakonov, 1961; Jewett et al., 2015). Smirnov (2013) has reported 30 nominal species in Russian Pacific seas belonging to either *Henricia* or the allied genus, *Aleutihenricia* Clark & Jewett, 2010. This number is based mainly on Djakonov’s publications (1948; 1950; 1958; 1961), Hayashi (1940), and the work by Clark & Jewett (2010). Hence, species abundance in Russian Pacific is still in need of study.

Comprehensive accounts of the Vostok Bay marine biota began following the construction of the Vostok Marine Biological Station in 1970. The station was established by the Soviet Academy of Sciences and was a significant research site used by many Russian and USSR biologists. The echinasterids of this region have been overlooked. In recent literature, the reviews on echinoderm fauna of Vostok Bay (Dautov & Tyurin, 2014) and of adjacent Far Eastern National Marine Reserve (Lebedev, 2015), this family was represented by “*Henricia* sp.*”* only. Earlier literature review of Peter the Great Bay biota (with relevant section based on Djakonov (1961) reported seven *Henricia* species (Adrianov & Kussakin, 1998). Thereby, the fauna of *Henricia* of Vostok Bay remains totally unknown except for a single *Henricia* sp. of 21 species potentially inhabiting the Sea of Japan (Smirnov, 2013). Recent studies reported detailed morphology of 13 species from the Sea of Japan (Shin & Ubagan, 2015a; Shin & Ubagan, 2015b; Ubagan & Shin, 2016; Chichvarkhin, 2017a; Chichvarkhin, 2017b; Chichvarkhin & Chichvarkhina, 2017a; Chichvarkhin & Chichvarkhina, 2017b) and adjacent waters of Yellow Sea (Xiao, Liao & Liu, 2011).

Many sea stars including the species of the genus *Henricia* are a popular objects in biochemical and biomedical research because of perspective drugs found in these animals (e.g.,  Fedorov et al., 2008; Utkina, 2009). Taxonomic confusions have prevented confident identifications for *Henricia*, which has limited potential applications for this research field. Recent studies of coastal marine benthic fauna of Russian Pacific shores revealed numerous previously overlooked species belonging to different taxonomic groups (e.g.,  Chichvarkhin et al., 2016; Ekimova et al., 2016; Temereva & Chichvarkhin, 2017), hence a discovery of new species in a poorly studied and confused group like the Echinasteridae in Vostok Bay remains possible. Therefore, this study aims to shed light to *Henricia* fauna occurring in Vostok Bay by delineating putative species by the means of DNA barcoding, then finding corresponding morphological characters that are diagnostic for identification.

## Material and Methods

**Figure 1:**
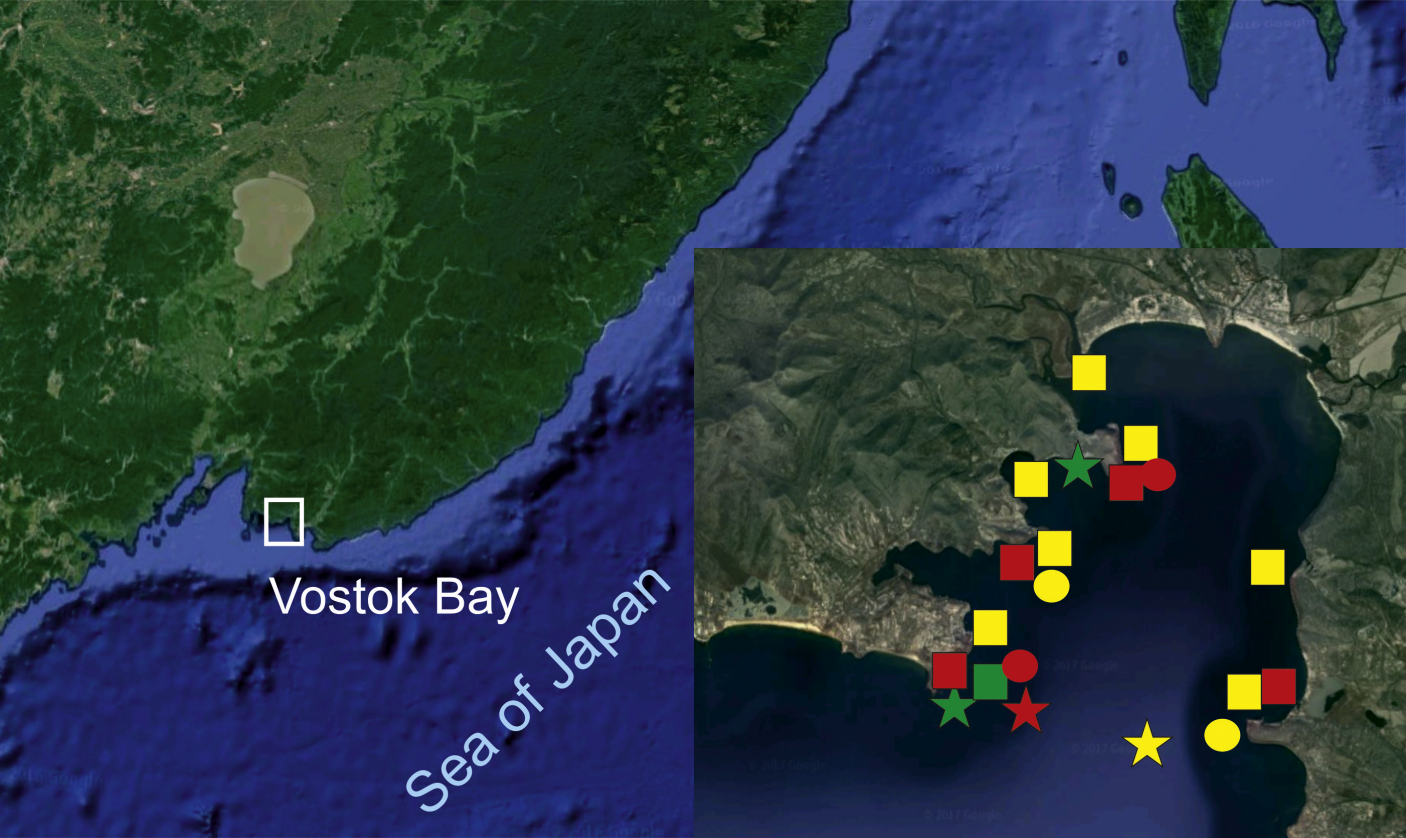
Surveyed area map (image by Google, 2017). SQUARES: yellow, *H. djakonovi*; red, *H. hayashii*; green, *H. granulifera*. CIRCLES: yellow, *H. nipponica*; red, *H. densispina*. STARS: yellow, *H. asiatica*; red, *H. pacifica*; green, *H. alexeyi.*

Observations and sample collections were made by SCUBA-diving in 2014 through 2017 in Vostok Bay of the Sea of Japan (Fig. 1). Most of the individuals belonging to abundant species were returned and released where each was collected. The images were taken with a Nikon D810 or D7000 cameras and a Nikkor 60/2.8 lens. Also, remotely controlled submersible apparatus Obzor-150 was used in 2015 at the depth of 45 m at the silty outer part of the bay. The specimens were preserved in 96% ethanol or dry with prominent portion of a ray preserved in separate vials in 96% ethanol and deposited in the Museum of National Science Center of Marine Biology, Russian Academy of Sciences (Vladivostok, Russia). Skeletal plates and spines were denuded using 5–15% sodium hypochlorite solution at room temperature. Scanning electron images of the spines were obtained using Zeiss Sigma and Zeiss Evo electron microscopes after carbon coating. Studied specimens are preserved in the collections of Zoological Institute of Russian Academy of Sciences, St. Petersburg, Russia (voucher code *ZIN*), National Science Center of Marine Biology, Russian Academy of Sciences (voucher code *MIMB*) and Hokkaido University, Sapporo, Japan (voucher code *ZIHU*). Sequenced specimens are listed in the Table 1.

**Table 1:** Species included in the study, their sampling location, voucher numbers and GenBank accession numbers of the 16S rRNA sequence.

Species	n	Voucher #	GenBank #, 16S
*Henricia alexeyi,* paratype	Vostok Bay	*MIMB-* 33244	KY464043^a^
*Henricia alexeyi*	Vostok Bay	*MIMB-* 34803	KY464042^a^
*H. alexeyi*	Rudnaya Bay	*MIMB-* 33243	MG976220^a^
*H. pacifica*	Vostok Bay	*MIMB-* 33247	KY464039^a^
*H. pacifica*	Vostok Bay	*MIMB-* 33246	KY464040^a^
*H. pacifica*	Vostok Bay	*MIMB-* 33248	KY464041^a^
*H. ohshimai*	Japan	*-*	DQ297093
*H. ohshimai*	Japan	*-*	AB084571
*H. densispina*	Vostok Bay	*MIMB-* 34800	MG976223^a^
*H. densispina*	Vostok Bay	*MIMB-* 34801	MG976224^a^
*H. densispina*	Vostok Bay	*MIMB-* 34801	MG976225^a^
*H. djakonovi*	Vostok Bay	*MIMB-* 33878	KY464037^a^
*H. djakonovi*	Vostok Bay	*MIMB-* 33128	MG976221
*H. djakonovi*	Vostok Bay	*MIMB-* 33831	MG976222
*H. djakonovi,* holotype	Rudnaya Bay	*MIMB-* 33129	KY464038
*H. hayashii*	Vostok Bay	*MIMB-* 33544	KY744471
*H. hayashii*	Vostok Bay	*MIMB-* 33250	KY744470
*H. granulifera*	Vostok Bay	*MIMB-* 33251	KY744469
*H. oculata*	Atlantic Ocean, UK	*-*	AY652500
*H. oculata*	Commander Is.	*MIMB-* 33214	KX986916^a^
*H. oculata*	Commander Is.	*MIMB-* 33215	KX986917^a^
*H. nipponica*	Vostok Bay	*MIMB-* 33252	KX610476^a^
*H. nipponica*	Vostok Bay	*MIMB-* 33253	KX610479^a^
*H. nipponica*	Vostok Bay	*-*	KX610478^a^
*H. nipponica*	Vostok Bay	*-*	KX610477^a^
*H. nipponica*	Japan	*-*	D63737
*H.* cf. *nipponica*	Japan	*-*	D63739
*H. tumida*	NE Pacific	*-*	AF290033
*H. sanguinolenta*	Norway	*-*	KT268115
*H. obesa*	Australia	*-*	KT268113
*H. compacta*	Australia	*-*	KT268112
*H. lineata*	Avacha Bay	*MIMB-* 33541	MF133325
*H. uluudax*	Avacha Bay	*MIMB-* 33543	KY934075
*H. olga,* holotype	Rudnaya Bay	*MIMB-* 33539	KY934079
*H.* sp.		*-*	HM473897
*H.* sp.		*-*	HM473896
*H.* sp.		*-*	HM473895
*H.* sp.		*-*	HM543156
*H.* sp.		*-*	DQ297093

**Notes.**

a Data obtained in this study.

DNA was extracted using the Diatom™ DNA Prep 100 kit (Isogene Lab, Moscow, Russia) according to the manufacturer’s protocol. Partial sequence for mitochondrial 16S rRNA gene (16S) was used in this study. This fragment was used as a good species-specific marker in several *Henricia* and echinoderm studies (Chichvarkhin & Chichvarkhina, 2017b). The primers 16Sar and 16Sbr (Palumbi, 1996) were used to amplify the region of interest. Polymerase chain reaction amplifications were carried out in a 20-µL reaction volume, which included 4 µL of 5×  Screen Mix by Eurogen Lab We also attempted unsuccessfully to amplify the portion of the mitochondrial gene for cytochrome *c* oxidase subunit I (COI) that is commonly used in metazoan DNA barcoding. The primers normally employed by Folmer et al. (1994) do not work well for echinasterids. We also attempted unsuccessfully to amplify the portion of the mitochondrial gene for cytochrome c oxidase subunit I (COI) that is commonly used in metazoan DNA barcoding. The master mix for each sample was prepared using 34.75 µL H_2_O, 5.00 µL PCR Buffer (Evrogen, Moscow, Russia), 5.00 µL 25 mM MgCl_2_, 1.00 µL 40 mM dNTPs, 1.00 µL 10 mM primer 1, 1.00 µL primer 2, 0.25 µL 5 mg/ml (10×) Taq (Evrogen, Moscow, Russia), and 1.00 µL extracted DNA. Amplification began with an initial denaturation for 1 min at 95 °C followed by 40 cycles of 15 s at 95 °C, 15 s at 52 °C, 30 s at 72 °C with a final extension of 7 min at 72 °C. Sequencing for both strands proceeded with the Big Dye v3.1 sequencing kit by Applied Biosystems. Sequencing reactions were analyzed using ABI 3130 and 3500 Genetic Analyzer (Applied Biosystems, Foster City, CA, USA) in National Scientific Center of Marine Biology (Vladivostok, Russia). The sequences were aligned with ClustalW software (Larkin et al., 2007). Tree-based methods for species delineation and identification were used, including the calculation of pairwise p-distances (i.e., the proportion of variable positions) and neighbor-joining (NJ) clustering using MEGA7 software (Kumar, Stecher & Tamura, 2016).

The primers normally employed by Folmer et al. (1994) do not work well for echinasterids. Recent works (Laakmann et al., 2016; Layton, Corstorphine & Hebert, 2016) have demonstrated that the COI can distinguish some entities in the Echinasteridae. Knott, Ringvold & Blicher (2017) used *Henricia*-specific primers more successfully: they delimited almost all north Atlantic species, although we failed to amplify any product using these primers. Often, the COI can be poorly or not amplified with ‘Folmer’s’ primers (Folmer et al., 1994) commonly used in metazoan DNA barcoding, hence we are considering the 16S is a more robust and preferable marker for routine DNA barcoding of Asteroidea (Chichvarkhin, 2017b; Chichvarkhin, 2017b; Knott, Ringvold & Blicher, 2017). Recently, Knott, Ringvold & Blicher (2017) have developed *Henricia-* specific primers to amplify the COI. Amplified fragment was same informative as the 16S, hence we see no reason to use it as a marker in our study.

In his descriptions of *Henricia* species, Alexander M. Djakonov (Djakonov, 1948; Djakonov, 1958) never designated type materials; the specimens that we identified in ZIN collection by the labels written by him and his co-worker Zoya I. Baranova, and collection dates preceding a publication. Certain of these syntypes series contain or may contain specimens belonging to different species, so we have fixed a particular syntype as the lectotype in such cases, with all other specimens in each series becoming paralectotypes.

For the taxa below, the author (Djakonov, 1958; Djakonov, 1961) did not designate unique specimens as the holotypes according to International Code of Zoological Nomenclature (ICZN) Article 73.1.3, i.e., the series of the syntypes were used for description. For all these species, we identified the specimens marked as the “holotypes” in ZIN collection. But this method for a holotype fixation applied by Z.I. Baranova is incorrect according to ICZN Article 72.4.7, since these specimens unequivocally belong to the type series according to ICZN Article 72.4.1.1. To avoid confusion, here we are fixing unique specimens as the lectotypes of several of Djakonov’s taxa based on the specimens used in this study. Appropriate lectotypes designations are given in species sections below. We disregarded ”holotype” labels later added to particular syntype specimens in the ZIN collection in the hand-writing of Djakonov’s assistant, Z.I. Baranova, because such holotype designations are invalid according to the International Code of Zoological Nomenclature (ICZN) articles 72.4.1.1, 72.4.7, and 73.1.3.

## Results

### Molecular analysis

**Figure 2:**
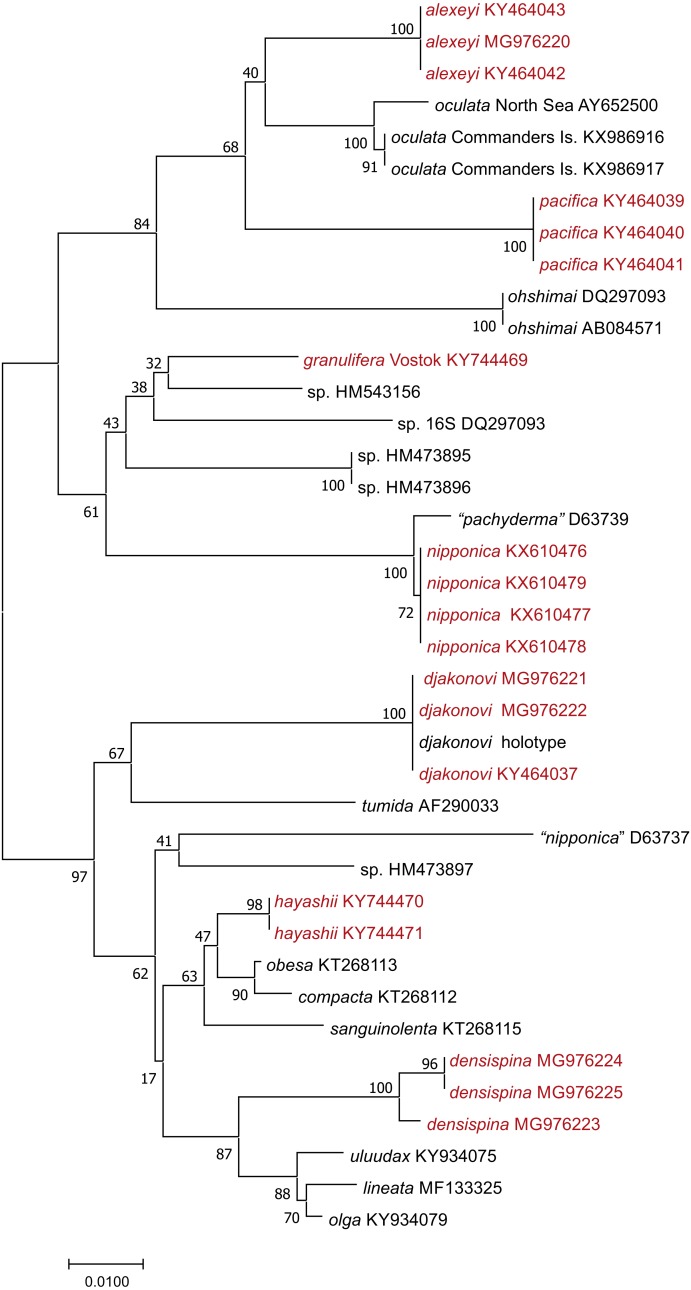
NJ tree (*p*-distance) based on 16S rRNA partial nucleotide sequences. Bootstrap 1,000 replicates. The taxa marked with red were colleccted in Vostok Bay.

We used 16S sequences from this study, or added from GenBank, were partial 16S rRNA nucleotide sequences ranging from 541–606 bp long. We produced an alignment of 632 bp including gaps; 162 sites within the ingroup were variable, and 129 of these were parsimony-informative. Neighbor-Joining tree (Fig. 2) shows well supported clusters for all were partial 16S rRNA nucleotide sequences ranging from 541–606 bp long. We produced an alignment of 632 bp. Uncorrected interspecific distances ranged from 5% (*H. lineata –H. uluudax)* to 11.5% (*H. ohshimai –H. compacta*).

**Table:** 

Order Spinulosida Perrier, 1884
Family Echinasteridae Verrill, 1870
Genus *Henricia* Gray, 1840

### Subgenus *Henricia* Gray, 1840

**Table:** 

*Spinohenricia* Heding, 1935
*Magdalenaster* Koehler, 1907
*Cyllaster* A.H. Clark, 1916

Type species: *Asterias oculata* Pennant, 1777

**Figure 3:**
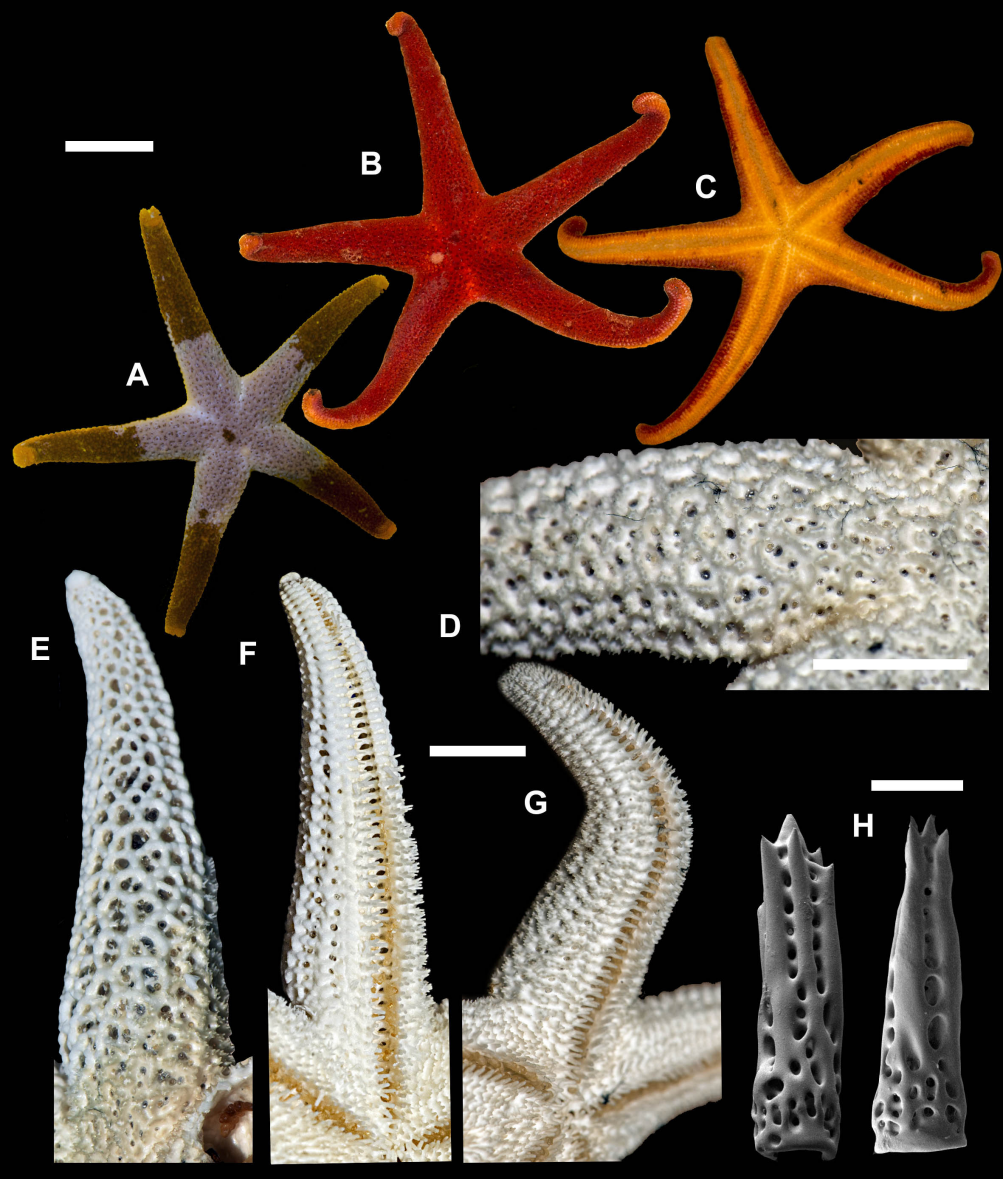
*Henricia alexeyi.* Chichvarkhin & Chichvarkhina, 2017. (A–C) live specimens, paratypes; (D, E) abactinal side; (F–G) actinal side; (H) abactinal spines. Scale bars: A–C, F–G - 10 mm, D - 5 mm, H - 50 µm. Photos by A. Chichvarkhin.

**1. *Henricia (H.) alexeyi* Chichvarkhin & Chichvarkhina, 2017a; Chichvarkhin & Chichvarkhina, 2017b (Fig. 3)**

**Table:** 

*Henricia alexeyi* Chichvarkhin & Chichvarkhina, 2017a: 203–207, figs. 1–3, 4G;
Chichvarkhin & Chichvarkhina, 2017b: 25–27, fig B, C.

Type locality: Rudnaya Bay, Sea of Japan, Russia.

Examined material: holotype: *MIMB*–*33243,* Senkina Shapka pinnacle, Rudnaya Bay, 10–15 m depth, 8 October 2015, leg. A. Chichvarkhin. Paratypes: *MIMB*–*33244*, 1 spm, Senkina Shapka pinnacle, Rudnaya Bay, 10–15 m depth, 8 October 2015, leg. A. Chichvarkhin; *MIMB-* 34803, 1 spm, Vostok Bay, 07 Jul 2017, leg. K. Dudka; *MIMB*–*33241,* 1 spm, Vostok Bay, Sea of Japan, Russia, Aug 2015, leg. K. Dudka; *MIMB–33242,* 1 spm, Vtoraya Priboika Inlet, Vostok Bay, Sea of Japan, Russia, 2 m depth, 25 Aug 2016, leg. I. Ekimova. *ZIHU*–*2405*, 3 syntypes of *H. ohshimai* (Hayashi, 1940), Onagawa shore, 1934.

External anatomy. Small size sea stars, R (long radius, mouth to ray tip) = 19–47 mm, r (short radius, mouth to interradius) = 4–9 mm, mean R: *r* = 4.7 (R:r of holotype 4.56, *R* = 20.5, *r* = 4.5). Rays five, cylindrical, slim, not swollen at base (Fig. 3). Abactinal skeleton open meshed. Abactinal plates oval, rod-shaped with low ridges or tubercles. Papular areas with 1–3 papulae. Abactinal pseudopaxillae (Fig. 3B) with 6–10 spines in two rows covered by thin skin. Abactinal spines short, stout, 300 µm long, 80–100 µm wide, most tapering distally (Fig. 3), bear 1–6 short thick subapical thorns faced upwards.

Superomarginal row well discernible consisting of plates larger than abactinal, with rather high ridges (Figs. 3A, 3C), consists of longitudinally elongated plates. Intermarginal row extended to 2/3 of ray length, second intermarginal row short, consists of eight plates. Intermarginal plates small, round. Inferomarginals plates triangular or sub-quadrate. Superomarginals bear 7–9 spines in two rows. Intermarginal pseudopaxillae with 4–6 spines in two rows. Inferomarginal pseudopaxillae bear 10–12 spines in 2 transversal rows.

Ventrolateral row consists of densely set oval plates. Second ventrolateral row extends to a half of ray length, consists of tiny round plates. Ventrolateral pseudopaxillae bear 3–5 spines in one row. Adambulacrals with 3–4 larger near-furrow spines and three small spines in one irregular row (Fig. 3C, 3D) joined by skin membrane as a crest. Proximally, adambulacral, ventrolateral and inferomarginal spines arranged in crests with skin membrane (Fig. 3D). Deep furrow spine small, single. Oral plates each bear single apical, three marginal, and two suboral spines.

Color in life varies greatly from red to orange. Some specimens with broad darker areas on rays or almost white on disc and proximally on rays (Fig. 3). Anal area darker or same color as background; madreporite lighter or same color as background. Actinal side uniformly orange.

Ecology: inhabits open bays exposed to wave activity at 2–15 m depths. In Rudnaya Bay, specimens were found on sponges apparently damaged by these sea stars (Fig. 3F).

**Figure 4:**
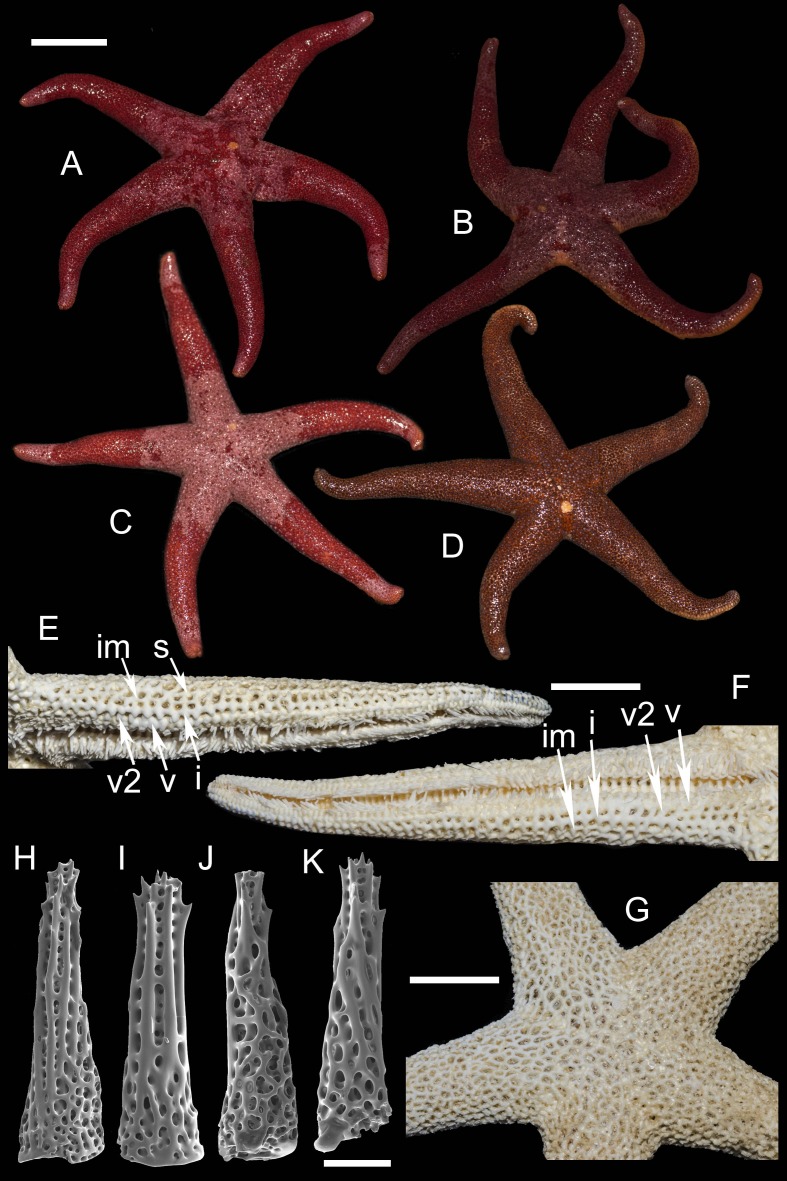
*Henricia pacifica.* (Hayashi, 1940). (A) holotype life coloration; (B–D) paratypes; (E, F) actinal skeleton; (G) abactinal skeleton; (H–K) abactinal spines. i, inferomarginal; im, intermarginal; s, superomarginal; v, ventrolateral; v2, additional ventrolateral. Scale bars: A–D - 10 mm, E–G - 5 mm, H–K - 50 µm. Photos by A Chichvarkhin.

**2. *Henricia (H.) pacifica* Hayashi, 1940 (Fig. 4)**

**Table:** 

*Henricia pacifica* Hayashi, 1940: 152–154, Pl. 9 figs. 7–10; Hayashi, 1952: 114;
Djakonov, 1961: 16–17. (part.); Chichvarkhin & Chichvarkhina, 2017a: fig. 4H; Chichvarkhin & Chichvarkhina, 2017b: 27, fig D.

*Henricia sanguinolenta*—Yavnov, 2010: 125–126 (non Müller, 1776).

Type locality: Tsugaru Strait, Japan.

Examined material: *MIMB–33245,* (*R* = 46 mm) Vostok Bay, 10–15 m depth, Aug 2015, leg. I. Krasilnikov. 3 spms, *MIMB–33246–33248,* (*R* = 38, 48, 51 mm), Vostok Bay, 10–15 m depth, Aug 2015, leg. I. Krasilnikov; *ZIHU*–*2404, H. pacifica* holotype, Tsugaru Strait, Japan.

External anatomy. Medium size sea stars, mean R: *r* = 4.1 (R:r of holotype 4.2); *R* = 48–53 mm, *r* = 11–14 mm, R of holotype 50 mm, *r* = 12 mm. Rays five, slender, slim, cylindrical (Figs. 4A–4D). Abactinal skeleton open meshed. Abactinal plates oval, rod-shaped with well discernible smooth ridges (Fig. 4G). Abactinal spines set along ridges in groups of 4–10 spines in one row covered by skin membrane, so in dried specimens the spines become poorly distinguishable. Papular areas with 1–3 papulae. Abactinal spines long, tapering, 400 µm long, 100 wide at base, tapering to 30–50 µm distally (Figs. 4H–4K), bear 9–11 tiny thorns on apex and spine shaft and faced upwards or at angle to spine axis.

Superomarginal row discernible consisting of cross-shaped plates similar in size to neighboring plates with no ridges (Figs. 4E–4F). Intermarginal row extended to 2/3 of ray length. Intermarginal plates rod- and crescent-shaped. Inferomarginals plates sub-quadrate. Superomarginals bear 4–5 spines in one row. Intermarginal pseudopaxillae with 4–5 spines in two rows. Inferomarginal pseudopaxillae bear five spines in one transversal rows.

Ventrolateral plates sub-quadrate, smaller than inferomarginals. Pseudopaxillae bear 4–5 spines in one row. Adambulacrals with three larger thick near-furrow spines and four smaller spines in one row. Deep furrow spine thick, single. Oral plates each bear single apical, three large marginal, and three suboral spines.

Color, in life, bloody-red with light whitish tint proximally on rays and on disc, also on ray tips in some specimens (Figs. 4A–4D). Anal area not discernible from background; madreporite yellow. Actinal side uniformly orange.

Ecology: inhabits open bays exposed to wave activity at 15 m depths.

**Figure 5:**
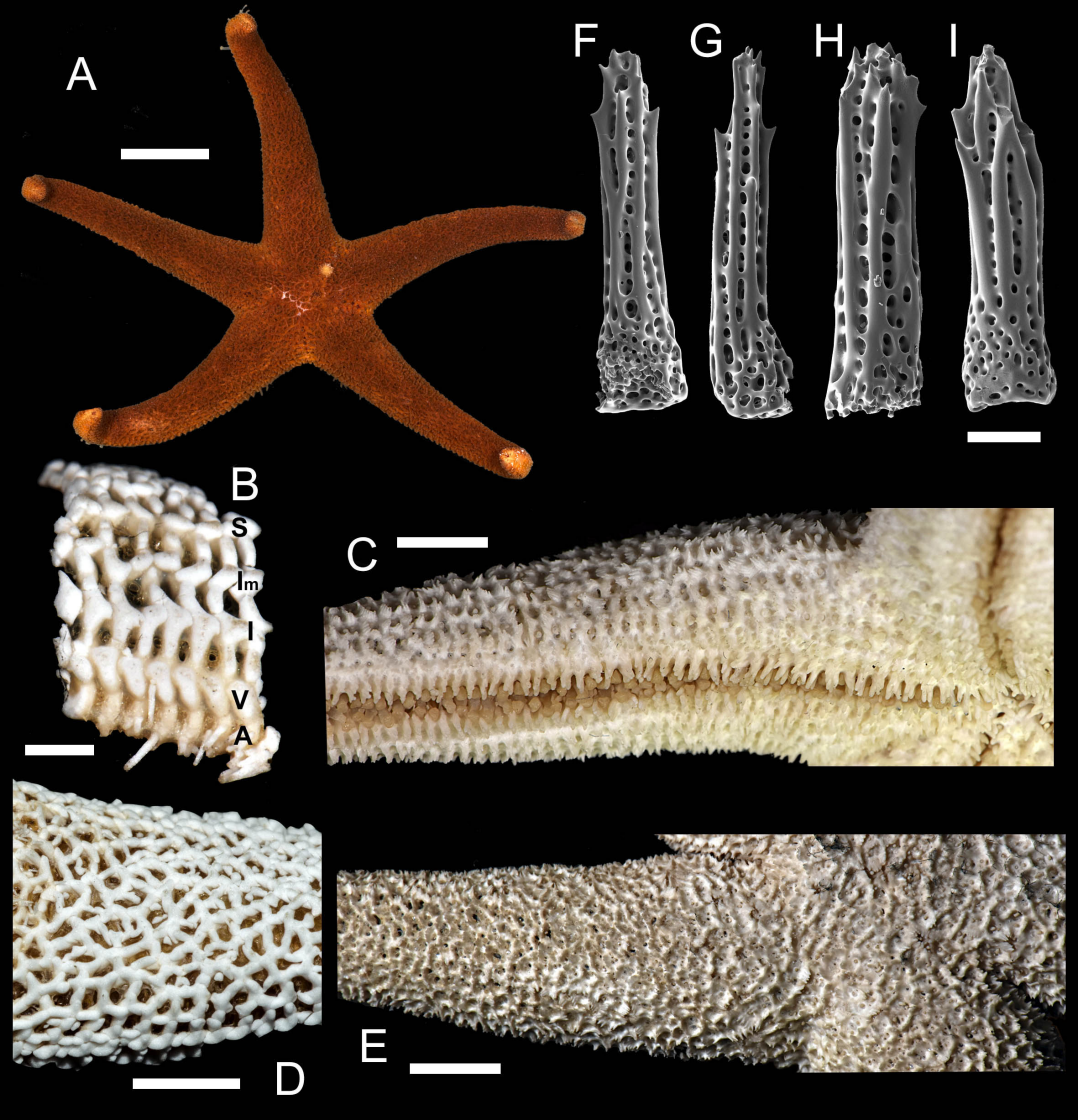
*Henricia nipponica.* Vostok Bay. (A) holotype, life coloration; (B) holotype, denuded acinal side; (C) holotype, actinal side of ethanol-preserved specimen; (D) holotype, denuded abactinal side; (E) holotype, abactinal side of ethanol-preserved specimen; (F–I) abactinal spines. i, inferomarginal; im, intermarginal; s, superomarginal; v, ventrolateral; a, adambulacral. Scale bars: A - 10 mm, B - 1 mm, C–E - 5 mm, F–I - 50 µm. Photos by A. Chichvarkhin.

**3. *Henricia (H.) nipponica* Uchida, 1928 (Fig. 5)**

**Table:** 

*Henricia leviuscula* var*. nipponica* Uchida, 1928: 794, pl. 32, figs. 6, 7.
*Henricia nipponica* Hayashi, 1940: 146–149, fig. 32–35.

*Henricia pachyderma,* Chichvarkhin & Chichvarkhina, 2017a: fig 4I; Chichvarkhin & Chichvarkhina, 2017b: 27, fig. E. (non Hayashi, 1940)

Type locality: Japan.

Examined material: 6 spms, *MIMB–33252*–*33257*, Vostok Bay, July 2015, leg. I. Krasilnikov; 1 spm, *MIMB–33814*, 4 Oct 2015, A. Chichvarkhin; 5 spms, Vostok Bay, 27 Aug 2016, leg. A. Goloseyev; 1 spm, Vostok Bay, 10 Jul 2017, leg. K. Dudka; 4 spms, Rudnaya Bay, 10 Oct 2015, leg. A. Chichvarkhin; 1 spm Sagami Bay, Misaki Marine Biological Station, Kanagawa, Japan, 3 Jun 2018, leg. A. Chichvarkhin.

External anatomy. R: *r* = 4.4–5.7, *R* = 35–50 mm. Rays five, abnormally six, cylindrical (Fig. 5A). Aboral skeleton wide meshed. Most abactinal plates rod-shaped, some branched with well discernible tubercle. Secondary plates of irregular shape in some meshes (Figs. 5B, 5D). Abactinal pseudopaxillae with 4–8 spines in one row. Papular areas with 1–3 papulae (most often three); papulae in live individuals large, 4–5 fold longer than wide (Fig. 5E). Abactinal spines long, 400 µm long, 60–100 µm wide at base, gradually tapering proximally (Figs. 5F–5I), with 8–12 tiny apical and subapical thorns, covered by skin membrane.

Superomarginal row poorly discernible, consists of plates similar to adjacent aborals, bearing 3–5 spines, same as abactinal spines, arranged in one row. Intermarginal row extended to a half of ray length; plates with 4–5 spines in one row. Plates of intermarginal and adjacent marginal rows connected by additional transversal plates. Inferomarginal plates large, transversally elongated, pseudopaxillae bear 8–10 spines in one transversal row.

Ventrolateral plates pillow-shaped, with proximal outgrowth overlaying next proximal plate; pseudopaxillae bear 4–7 spines in one transversal row. Second ventrolateral row consists of smaller round plates bearing 2–3 spines; this row extends to a half of ray length. Adambulacrals with 3–4 larger near-furrow spines and 6–8 smaller spines in two irregular rows (Fig. 5C). Deep furrow spine small, single. Oral plates not fused, each bear single apical, four marginal, and four suboral blunt spines.

Color in life. Abactinal side dirty-orange to brown, actinal side orange, lighter than abactinal side (Fig. 5A). Anal area darker than background; madreporite light creamy.

Ecology: The species was found on rocky substrates at the depths of 1–20 m at water temperature of 2–22° C .

Distribution. Abundant in the northern part of Japan (Hayashi, 1940). Reported here from Russian waters for the first time.

**4. *Henricia (H.) oculata robusta* Djakonov, 1958**

**Table:** 

*Henricia oculata:* Jewett et al., 2015: 76, 77.
*Henricia aspera*–Djakonov, 1948: 30; 1950: 99–100; 1961: 24–25 (non Fisher, 1911).
*Henricia aspera robusta:* Djakonov, 1948: 99, fig. 193; 1950: 99; 1958: 310–311; 1961: 25;
Yavnov, 2010: 177, 179 (part.); ? Xiao, Liao, Liu, 2010: 5, figs. 4A–F, 12G.

Type locality: southern Kurile Islands, Pacific shore.

Examined material: 1 spm (*H. oculata* neotype) *NHMD*-71387 (AMUC-AST-223), Treaddur Bay, Ravensport, 15 May 1968, leg. B. Brunn; 2 spms, *NHMD*-204701, Bay Fire, Isle of Man, Irish Sea, 24 Apr 1968, leg. B. Brunn; 2 spms, *NHMD*-76378 (ZMUC-AST-214), Plymouth, ca. 50 m, Engelska Kanal, Sep 1931, leg. Th. Horbensen; 1 spm (*NHMD*), Foró, >1,867 m, 8 Apr 1984, leg. Rostrup; *ZIN–15/15340*, 2 spms, Korea-Sakhalin Expedition st. 2, 1 Mar 1900, Peter the Great Bay, Bosphorus Eastern Str., 18 m, silt; *ZIN–8/15043*, 1 spm, Rossinant st. 1555, 3 Sep 1930, 83–86 m, Vladimir Bay, leg. Derjavin; *ZIN–26/15052*, 2 spms, Toporok st. 2, 16 Aug 1948, Musasi Bank, Sea of Japan, 93–96 m, leg. Ushakov; *ZIN–10/15156*, 2 spms, Nagasaki, 1901, leg. P.Yu. Schmidt; *ZIN–18/15343*, 2 spms, Nagasaki Apr 3, 1897, D-r Bunge; ZIN–17/15342, 2 spms, Nagasaki, Dec 12–14 1897, D-r Bunge; *ZIN–16/15341,* 1 spm, 23 Jul 1898, Nagasaki, D-r Bunge; *ZIN–14/15339,* 1 spm, 10–-15 Dec 1897, Nagasaki, D-r Bunge.

Diagnosis. Aboral pseudopaxillae comprised of up to 10–20 spines in double row. Spines covered with thick skin membrane. Spines stout, crowned with 15–20 tiny thorns, lateral ribs poorly developed. Abactinal plates arched, suboval, with no ribs, arranged in relatively wide mesh with 1–3 papulae a mesh. Superomarginals larger than aboral plates, T-shaped, with central proximal outgrowth. Inferomarginals large, cross-shaped, elongated transversally. Adambulacral spines 5–6, largest one near furrow spatulate or slightly flattened.

External anatomy: R:r 3.7–4.1 R 45–57 mm, r 11–15 mm. The rays slender not very slim, slightly swollen at base. Whole body covered by thick skin membrane, which looks rather thin in dried specimens. Abactinal plates suboval, rod-shaped, arched, cover adjacent proximal plates (Fig. 1A). Abactinal pseudopaxillae (Fig. 1B) crowned with 10–20 spines arranged in two rows. Papular areas nearly same width/length as average abactinal pseudopaxilla, includes one to three papulae. Abactinal spines covered with thin skin membrane, vary in length, stout, 350–400 µm long, 100 µm wide at base; apex with 15–20 short thorns, lateral ribs poorly developed (Fig. 2); in some individuals, spines short and widened distally, in the others, spines slender and tapering at distal end (Figs. 2A–2C).

Superomarginal row bent dorsally at ray base (Fig. 1C), consists of T-shaped plates with central dorsal outgrowth overlapping adjacent abactinal plates; bear 8–10 spines arranged in 2 rows. In large specimen, two complete or qincomplete intermarginal rows consisting of rod-shaped plates that connected to adjacent marginal rows with similar rod-shaped transversal plates crowned with 6–8 spines in two rows; one of intermarginal row extended to or beyond half ray. In this way, supero- and inferomarginal rows in some specimens (e.g., *MIMB-33214,* Fig. 1C) are widely separated with two intermarginal rows and two series of additional transversal plates. But in most specimens (Fig. 3D) intermarginal rows and connecting plates are less developed. Inferomarginal row extended over entire ray length, its pseudopaxillae bear 14–20 spines in two transversal rows. Ventrolateral quadratic plates bear 10–13 relatively small spines in 2–3 rows. Second ventrolateral row consists of about 18 transversally elongated rod-shaped plates with low tubercle. Arched adambulacrals with slightly curved small furrow spine, one large slightly flattened to spatulate spine (Figs. 1C, 1D) faced into furrow (but in some individuals, more than one spine is spatulate, Fig. 3F); the other 3–5 gradually smaller spines faced ventrally in one staggered row bearing numerous thorns on apices. Spatulate shape varies because it is constituted by soft tissue, not spine itself. Oral plates not fused, bear 4 marginal and 1–4 suboral spines.

Life coloration varies. Rays orange-red, pink, yellow, some individuals with purple to violet stripes (Figs. 2E, 2F). Madreporite slightly lighter than background.

Ecology. Living sea stars were found off Mednyi Island on rock at depths 5–10 m at water temperature of 4 °C. Probably, they fed on yellow colored sponge, which was detected nearby (Figs. 2E, 2F). In other areas, found at depths >100 m feeding on sponges, detritus, hydrozoans, and bryozoans (Jewett et al., 2015); brooding (Jewett et al., 2015).

Distribution. Amphiboreal species. NE Atlantic: British Islands, Irish Sea, English Channel, North Sea, Faroe Islands, France, Portugal, NW Atlantic: New Brunswick, northern Pacific: British Columbia, Alaska, Aleutian Islands, Commander Islands, Kurile Islands, Sea of Okhotsk, Sea of Japan, Pacific shore of Japan (Hayashi, 1940; Djakonov, 1961; Madsen, 1987; Clark & Jewett, 2010; Jewett et al., 2015). Unknown from Japan: the only specimen found in R. Hayashi’s collection preserved in Hokkaido University belongs to *H. aspera* Fisher, 1906.

### Subgenus *Aleutihenricia* (Clark & Jewett, 2010)

Type species *Henricia rudis* Verrill, 1911 (= *H. derjugini* (Djakonov, 1950)

**Figure 6:**
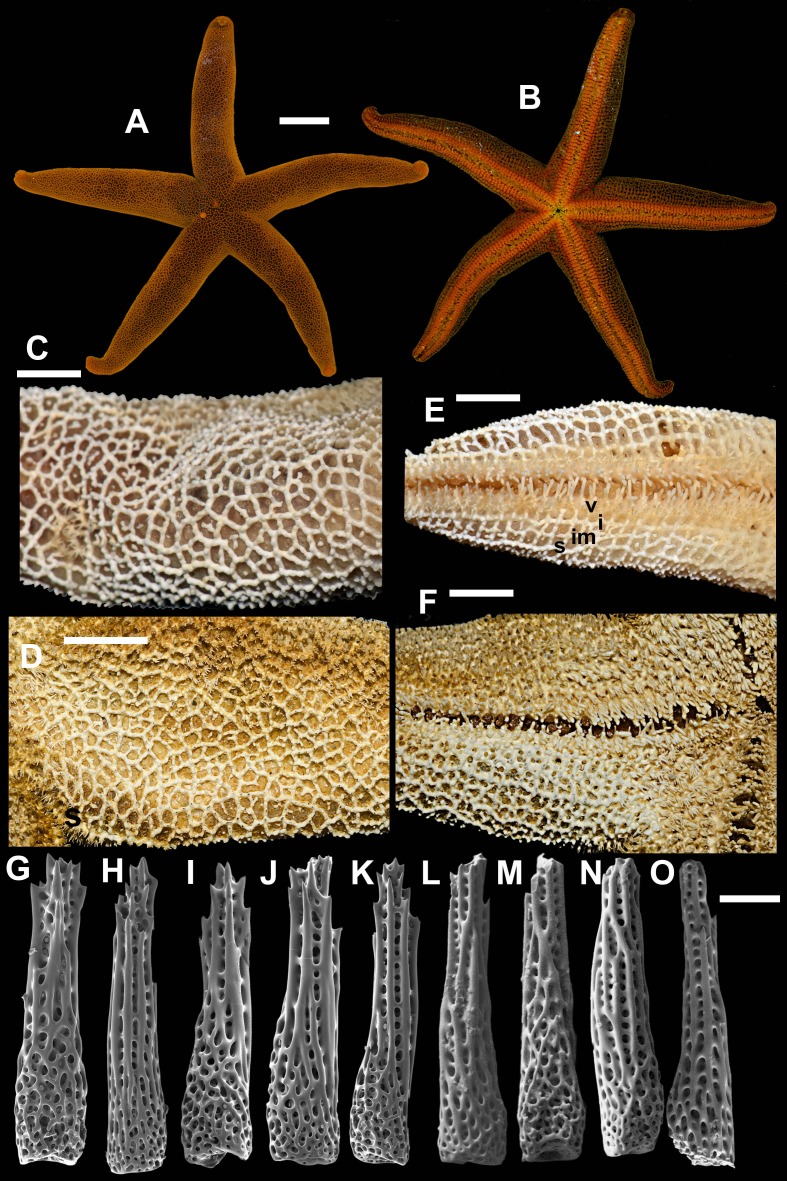
*Henricia granulifera,* Vostok Bay. (A, B) life coloration of abactinal and actinal sides, Vostok Bay; (C) denuded abactinal skeleton, Vostok Bay; (D) denuded abactinal skeleton, holotype; (E) actinal side, Vostok Bay; (F) actinal side, lectotype; (G–K) abactinal spines, Vostok Bay; (L–O) abactinal spines, *H. granulifera lect* otype. i, inferomarginal; im, intermarginal; s, superomarginal; v, ventrolateral. Scale bars: A, B - 10 mm, C–F - 5 mm, G–O - 50 µm. Photos by A. Chichvarkhin.

**5. *Henricia (A.) granulifera* Djakonov, 1958 (Fig. 6; Supplemental Information 1, second sea star, 5–9th seconds)**

We identified the specimen in ZIN collection (Fig. 6) identified as *H. granulifera* and labeled as the ‘holotype’ by Z.I. Baranova. We are designating this specimen as lectotype of *Henricia granulifera* (Djakonov, 1958). *R* = 80 mm, *r* = 19 mm; *ZIN*–*4/14824* transect off Moibesi [Belinskoye, W Sakhalin, Sea of Japan], 30–37 m, 30 Aug 1949.

*Henricia granulifera* Djakonov, 1958: 311–312, figs. 21, 22; Djakonov, 1961: 27, Pl. 2, fig. 20, Pl. 17, figs. 68, 69; (Yavnov, 2010): 121–122; (Chichvarkhin & Chichvarkhina, 2017a): fig 4K; Chichvarkhin & Chichvarkhina, 2017b: 27, fig. F.

Type locality: Sea of Japan, Tatar Strait near Belinskoye, Sakhalin.

Examined material: 1 spm, *MIMB–33251*, Vostok Bay, July 2015, leg. I. Krasilnikov; 1 spm. Vostok Bay, 13 July 2017, leg. K. Dudka; 1 spm, *ZIN*–*4/14824* (lectotype of *H. granulifera*) transect off Moibesi [Belinskoye, W Sakhalin, Sea of Japan], 30–37 m, 30 Aug 1949, Toporok stations #65–66; *ZIHU–2411* (holotype of *H. reticulata*), Onagawa Iigohama, Japan; *ZIHU–* 2412 (holotype of *H. irregularis*), Yezo Strait, Albatross 1906 station #5031, 86 fathoms depth, Japan.

External anatomy. Relatively large sea star, R 72 mm, *r* = 9, R: *r* = 8. Rays five, abnormally four, cylindrical. Disc small, aboral skeleton wide meshed. Abactinal plates rod-shaped, with 1–2 well discernible tubercles on each, form wide mesh. Small secondary plates of irregular shape in some meshes. Abactinal pseudopaxillae in groups of 3–5 spines in paxilla. Papular areas with 2–4 papulae. Abactinal spines long, 550 µm long, 100 µm wide at base, gradually tapering proximally (Figs. 6H–6P), with about 15 tiny and subapical thorns, some thorns located laterally on spine shaft; lateral thorns faced at an angle to spine shaft; spines not covered by skin membrane.

Marginal rows poorly discernible, consists of similar plates, bearing 3–5 spines; plates with well discernible tubercles. Superomarginal plates higher than adjacent plates, form more discernible bent row. Intermarginal row extended to full ray length. Inferomarginal plates large but not as high as superomarginals.

Ventrolateral plates small, round, with tubercle; bear 5–6 spines in a circle. Adambulacrals with 2–3 larger near-furrow spines and 5–6 smaller spines in two rows (Figs. 6C–6D). Deep furrow spine small, single. Oral plates not fused, each bear single apical, four marginal, and three suboral blunt spines.

Color in life. Abactinal side brown with spine apices orange (Fig. 6A); marginal rows brown as aboral side; ventrolateral, adambulacral, oral and furrow areas orange (Fig. 6B). Anal area small and lighter than background; madreporite bright orange.

Ecology. A single specimen of this species was found on rocky substrate at the depth of 15 m at water temperature of 16 °C.

Distribution. Known for the northwestern part of the Sea of Japan (Chichvarkhin & Chhichvarkhina, 2017), Korea (Shin & Rho, 1996) and Japan (Hayashi, 1940).

### Subgenus *Setihenricia* Chichvarkhin & Chichvarkhina, 2017

Type species *Henricia hayashii* Djakonov, 1961

**Figure 7:**
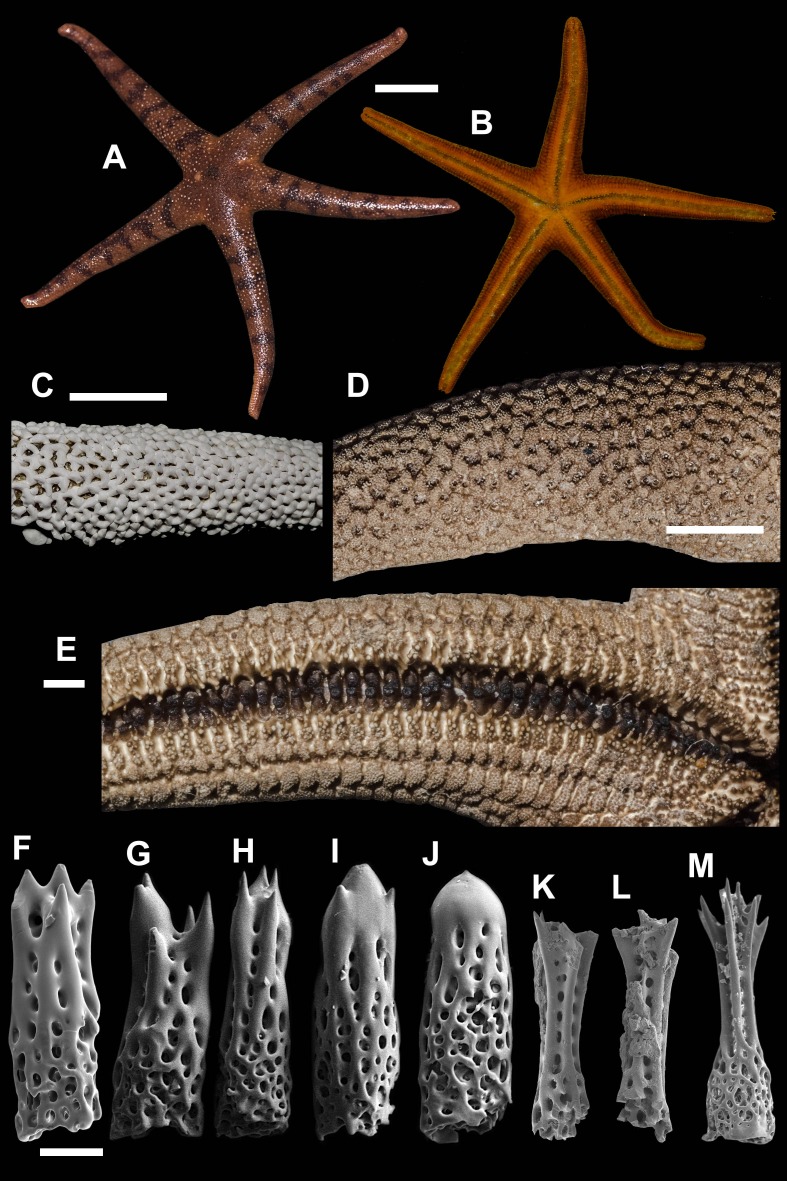
*Henricia djakonovi,* Vostok Bay. (A, B) life coloration; (C) denuded abactinal skeleton; (D) abactinal side of ethanol-preserved specimen; (E) actinal side of ethanol-preserved specimen; (F–H) abactinal spines; (I–J) abactinal spines, holotype; (K–L) abactrinal spines of *H. pseudoleviuscula,* holotype; (M) aboral spines of *H. reniossa,* holotype. Scale bars: A, B - 10 mm, C - 5 mm, D - 3 mm, E - 1 mm, F–M - 50 µm. Photos by A. Chichvarkhin.

**6. *Henricia (S.) djakonovi* Chichvarkhin, 2017 (Fig. 7)**

**Table:** 

*Henricia djakonovi* Chichvarkhin, 2017a: e2863; Chichvarkhin & Chichvarkhina, 2017a: Fig. 4E; Chichvarkhin & Chichvarkhina, 2017b: 27, fig. G.
*Henricia pseudoleviuscula*–Chichvarkhin, 2017a: e2863 (non Djakonov, 1958).
*Henricia hayashii* –Yavnov, 2010: 113–114 (non Djakonov, 1961).
*Henricia leviuscula leviuscula* –Xiao, Liao & Liu, 2011: 9–11, fig. 7 (non Stimpson, 1857).

Type locality: Rudnaya Bay, Sea of Japan, Russia

We identified *H. pseudoleviuscula* specimen in ZIN collection labeled as the “holotype” presumably by Z.I. Baranova. We are designating this specimen as lectotype of *Henricia pseudoleviuscula* (Djakonov, 1958). *R* = 41 mm, *r* = 8 mm; *ZIN*–*1/12815,* Toporok station 31, Moneron Is., 14 Aug 1947, 84 m depth.

Examined material: *MIMB–331294* 4 Jun 2016, Senkina Shapka pinnacle, Rudnaya Bay, 44.36°N 135.83°E, 16 m, leg. A. Chichvarkhin (holotype); *MIMB–331304* 4 Jun 2016, Senkina Shapka pinnacle, Rudnaya Bay, 44.36°N 135.83°E, 16 m, leg. A. Chichvarkhin (paratype); 11spms, *MIMB–33128, MIMB–33131*, 23–25 Aug 2015, Vostok Bay, leg. I. Krasilnikov; 1 spm, *MIMB–*, Vostok Bay, 10 Jul 2017, leg. K. Dudka; *ZIN*–*1/12815,* Toporok station 31, Moneron Is., 14 Aug 1947, 84 m depth (lectotype of *H. pseudoleviuscula*); 2 spms, *ZIHU*–*2397*, Bomasiri Shima [N. off Rebun Is., Japan], 1906 (*H. reniossa* holotype and paratype).

Chichvarkhin & Chichvarkhina (2017a) and Chichvarkhin & Chichvarkhina (2017b) described this species as one of two ‘tiger-striped’ sympatric morphs that co-occur in the northwestern Sea of Japan, while the other form was incorrectly referred to as *H. pseudoleviuscula*. This study found that both morphotypes had identical sequences, so they more likely represent discontinuous phenotypes of the same species (Fig. 2); both forms cannot be referred as *H. pseudoleviuscula* (Djakonov, 1961) because the lectotype of the latter possesses very different spines (Figs. 7K, 7L), similar to those of *H. reniossa* Hayashi, 1941 (Fig. 7M). Thus, our amended description of *H. djakonovi* here is expanded to include *H. pseudoleviuscula* sensu (Chichvarkhin, 2017a).

External anatomy. R of holotype = 52 mm, *r* = 11 mm, R: *r* = 4.7–5; *R* = 38. To avoid a future confusion, here we are re-describing *H. djakonovi* by combining the characters of both forms (i.e., *djakonovi* and *pseudoleviuscula* sensu Chichvarkhin, 2017a. mm, *r* = 7.5 mm in paratype. Rays slender not very slim, slightly swollen at base, tapering to blunt tips. Most abactinal plates (>50%) cross-shaped with two proximal outgrowths covering adjacent plates, their elevated proximal sides form crescent-shaped pseudopaxillae. The other plates triangular or irregular shaped, lacking proximal outgrowths, form round and oval pseudopaxillae. Plates convex without ridges or tubercles. Abactinal surface (Figs. 7A, 7C, 7D) thick, rigid; abactinal plates on disc close-set forming tight reticulation. Abactinal pseudopaxillae bear 20–30 spines, but some smaller pseudopaxillae bear 8–10 spines. Papular areas nearly twice smaller than average abactinal pseudopaxillae, include one or two papulae. Abactinal spines short, stout, 200–240 µm long, 60–100 µm wide, barrel-shaped (Figs. 7F–7J). Apex of most spines massive with few short thorns; apex in some specimens smooth, resembles glass droplet (Fig. 7J).

Superomarginal row bent dorsally at ray base, consists of square pillow-shaped plates. Inferomarginals and ventrolaterals are similarly pillow-shaped. Intermarginal rows consisting of usually one, but up to six, irregular shaped plates, constituting a proximal intermarginal area that provides an inflation of ray base. Superomarginals bear 20–35 spines, same as abactinal spines, arranged in four rows. Inferomarginal row extended over entire ray length (Fig. 7B), its pseudopaxillae bear 30–45 spines in 4–6 transversal rows.

Ventrolateral pseudopaxillae bear 25–30 thorny spines in 4–5 rows. Adambulacrals with 10–11 spines in two transversal rows bearing numerous small thorns on apices; 3–4 larger adambulacrals near furrow spines; size of the other spines facing ventrally gradually decreases outwards from the furrow (Fig. 7E). Deep furrow spine small, single. Oral plates not fused, each bear single apical, four marginal, and four suboral blunt spines.

Color in life. Rays brownish-orange, abactinal side of the disc dark (Fig. 7C). Anal area almost black; madreporite partially creamy, partially brownish. In ethanol-preserved specimens, the madreporite is the same color as abactinal side of disc. Abactinal side of rays marked with dark brown to black spots of irregular shape, some of them look like broad transversal lines. Actinal side is uniformly colored orange-red, same as abactinal background color of rays.

Ecology. The species was found on rocky substrates at the depths of 1–20 m at water temperature 2–22 °C. In aquarium conditions, some were (one was?) kept over 10 months in an aquarium without any macroscopic food supply, which could indicate that they survived by grazing microscopic food, such as encrusting protists or diatoms.

Distribution. Known for the northwestern part of the Sea of Japan from Peter the Great Bay to Oprichnik Bay. Also, reported from Yellow Sea as *H. l. leviuscula* (Xiao, Liao & Liu, 2011). A species, *H. pseudoleviuscula* Djakonov, 1958, with similar skeleton pattern lacking the ridges on the plates had been described from Moneron Island in the northwestern Sea of Japan. Life color of this species remains unknown but aboral spines in *H. pseudoleviuscula* is similar to the spines of type specimens of *H. reniossa* Hayashi, 1940, that are rather different from *H. djakonovi* (Figs. 7K–7M).

**Figure 8:**
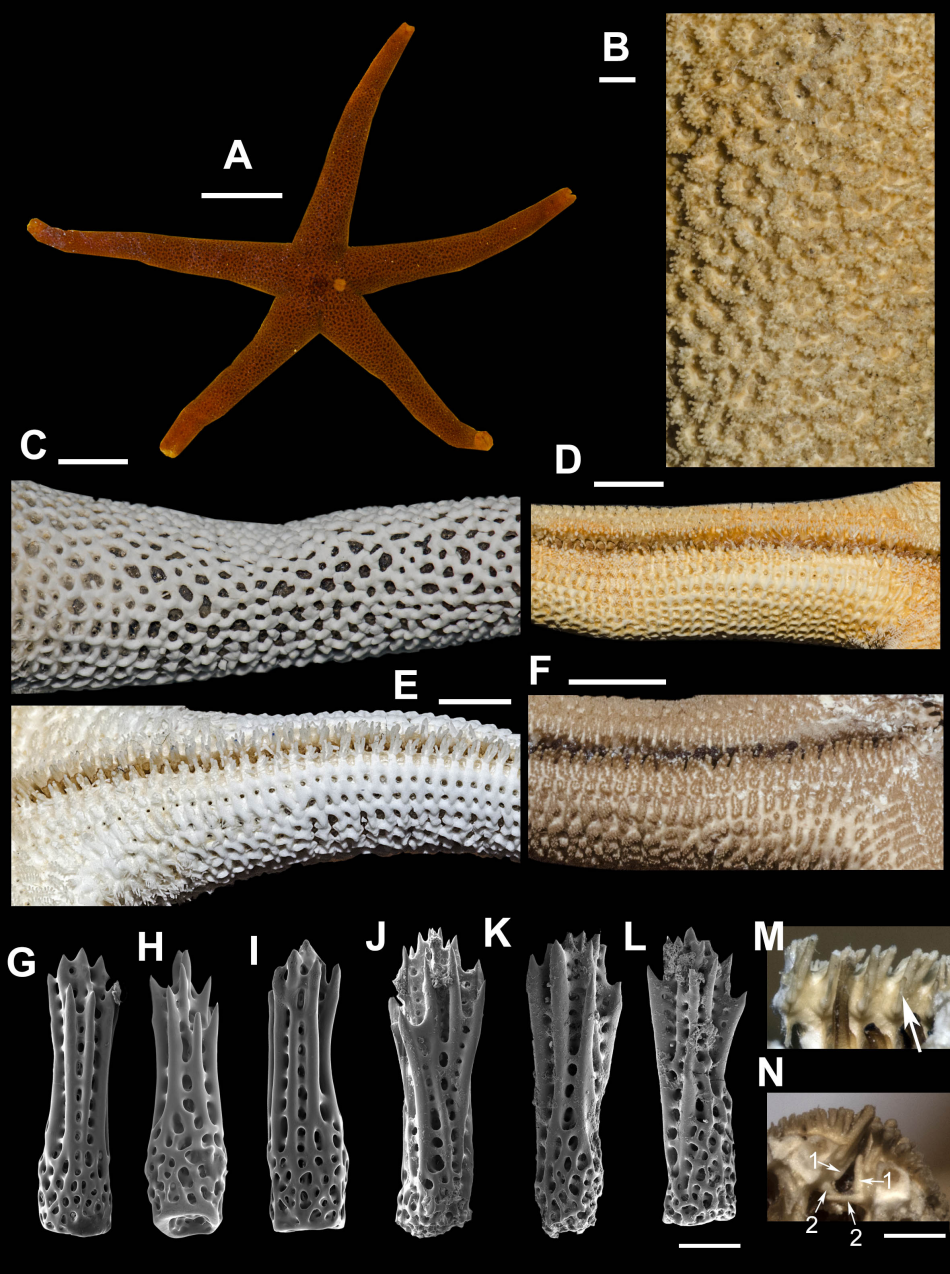
*Henricia hayashii*. (A) life coloration, Vostok Bay; (B) abactinal side, lectotype; (C) denuded abactinal side, Vostok Bay; (D) actinal side, lectotype, (E) denuded actinal side, Vostok Bay; (F) actinal side of ethanol-preserved specimen; (G–J) abactinal spines, Vostok Bay; (K–L) abactinal spines, holotype; (M) single deep adambulacral spines in the specimen from Peter the Great Bay, *ZIN*–*7/15940*; (N) double deep adambulacral spines in the specimen from Moneron Is., Scale bars: A - 10 mm, B - 1 mm, C–F - 5 mm, G–L - 50 µm, M, N - 500 µm. Photos by A. Chichvarkhin.

**7. *Henricia (S.) hayashii* Djakonov, 1961 (Figs. 8A–8N)**

*Henricia hayashii* Djakonov, 1961: 22–23, Pl. 2, fig. 8, Pl. 13, figs. 54–55); Chichvarkhin & Chichvarkhina, 2017a: fig4A; Chichvarkhin & Chichvarkhina, 2017b: 27, fig. H.

? *Henricia pseudoleviuscula* –Yavnov, 2010: 139–140 (non Djakonov, 1958.

Type locality: Sea of Japan, S off Povorotnyi Bay, Russia.

Examined material: *ZIHU*–*2938* (holotype of *H. reniossa* f. *tohokuensis* Hayashi, 1940, Soyomaru 1930 station 630, off Tobisima, 200 m depth, 10 Aug 1930; *ZIHU*–*2401* (holotype and paratypes of *H. saghaliensis* Hayashi, 1940, 3 broken spms, Albatross 1906, station 5023 Cape Patience, Sakhalin; 15 spms, *MIMB–33250*, Vostok Bay, Jul 2015, leg. I. Krasilnikov; 9 spms, Vostok Bay, 21 Aug 2016, leg. I. Krasilnikov; 5 spms Rudnaya Bay, 6 Jun 2016, leg. A. Chichvarkhin; *H. hayashii* det. A.M. Djakonov: 1 spm, *ZIN*–2/*14793*, KSE, Toporok st. #117, 22 Sep 1949, Nemoro Sea, 57 m depth, sand, rocks; 1 spm, *ZIN*–*5/15938*, Taimyr [vessel], 17 Oct 1812, La Perouse Str, Redun-to Isl., 45°20′N 141°31′E, 40 m depth; 1 spm, *ZIN*–*3/8863*, Sea of Japan, st. 219, 1912, leg. Pavlenko; 1 spm, *ZIN*–*4/6038* (marked as paratype of *H. hayashii*), TIRKh #1521, trawl #1, 21 Aug 1930, Sea of Japan, S off Povorotnyi Bay, 44 m depth, leg. Shurin; 1 spm, *ZIN*–*8/8629,* Far-Eastern expedition of Zool. Inst. Ac. Sci., st. 37, 8 Sep 1934, Sea of Japan, Sioukhu [Xiaohu?] Gulf; 1 spm, *ZIN*–*6/15939*, s/s Storozh st. 3, 20 Jun 1908, Sea of Japan, Askold Is., 25 m depth, on rocks, leg. Brazhnikova; 1 spm, *ZIN*–*7/15940*, s/s Storozh st. 6, 6 Jul 1908, Sea of Japan, Peter the Great Bay, 40-48 ftms depth, on rocks, leg. Brazhnikova; 1 spm, *ZIN*–*6/15939*, s/s Storozh st. 3, 20 Jun 1908, Sea of Japan, Askold Is; 1 spm, *ZIN*, Toporok st. #102, 6 Oct 1948, Moneron Is., 45 m depth, on gravel –more similar to *H. djakonovi* because of the absence of the ridges on intermarginal pates; 1 spm, *ZIN*–*4/6038* (lectotype of *H. hayashii*), south off Povorotnyi Cape, Sea of Japan, 21 Aug 1930, 44 m, leg. Shurin.

External anatomy. The specimens, found in Vostok Bay possesses *R* = 48–50 mm, *r* = 9 mm, R: *r* = 5.3–5.3. Rays five, cylindrical, slender. Aboral skeleton closely meshed. A bactinal plates (Fig. 8C) rod-shaped and triangular, tri-lobed, with low but discernible ridge. Abactinal pseudopaxillae reniform with 15–25 spines in two rows (Fig. 8B). Papular areas with 1–3 papulae. Abactinal spines (Figs. 8G–8M) long, 420 µm long, 100–120 µm wide at base, gradually extending proximally, with 10–15 apical and subapical thorns, not covered by skin membrane.

Superomarginal plates similar to adjacent aborals but slightly larger, bear 25–30 spines, arranged in 3–4 irregular rows. Intermarginal row extended to a third of ray length; plates round, with about 10 spines in one row. Inferomarginal plates large, with well discernible ridges transversally elongated, pseudopaxillae bear 15–21 spines in 2–3 irregular rows (Fig. 8C).

Ventrolateral plates small, oval; pseudopaxillae bear 10 spines in two irregular rows. Adambulacrals with four larger near-furrow spines and eight smaller spines in two rows (Fig. 8F). Deep furrow spine small, single, double at distal quarter of ray in some specimens. Oral plates each bear single apical, three marginal, and 1–2 suboral spines.

Color in life. Abactinal side bright orange, actinal side orange, lighter than abactinal (Fig. 8A). Anal area darker than background; madreporite light orange.

Ecology: The species was found on rocky substrate at the depth of 5–20 m at water temperature 16 °C.

Distribution. Known for the northwestern part of the Sea of Japan (Djakonov, 1961). Morphologically similar *H. reniossa asiatica* (Djakonov, 1958) was described from the northwestern shore of the Sea of Japan (Dzhygit Bay). Known specimens of this *H. reniossa asiatica* possess similar tightly meshed skeleton with distinct ridges on the inferomarginal plates as in *H. hayashii.* But it is significantly larger than any *H. hayashii* specimen that we ever studied (*R* = 142 mm), and possesses very unusually shaped aboral spines with blunt apex and a number of tiny denticles on it (Fig. 8N).

We identified the specimen imaged on fig. 54 of Djakonov (1961) in *Henricia hayashii* description in ZIN collection, it was labeled as the “holotype” presumably by ZI Baranova (Fig. 8). We found that this specimen does not possess double deep ambulacral spines (Fig. 8M) as described by Djakonov (1961). Also, we found a specimen identified by Djakonov as *H. hayashii* with two deep adambulacral spines (Fig. 8N) conforming the original diagnosis. It was the only one specimen with broken ray tip from Moneron Island, i.e., this is the only specimen in which deep spines might be counted earlier by Djakonov and/or Baranova: it is nearly impossible to observe these spines in an undamaged specimen. Therefore, we suppose that double deep adambulacral spine is not a distinctive character in this species, e.g., it is also specific for *H. densispina* (Sladen, 1878). All studied specimens from continental shore of the Sea of Japan possess single deep spine. Here, we are designating one of these specimens as the lectotype of *Henricia hayashii* (Djakonov, 1961): *ZIN*–*4/6038*, South off Povorotnyi Cape, Sea of Japan, Russia, 21 Aug 1930, 44 m, leg. Shurin.

**Figure 9:**
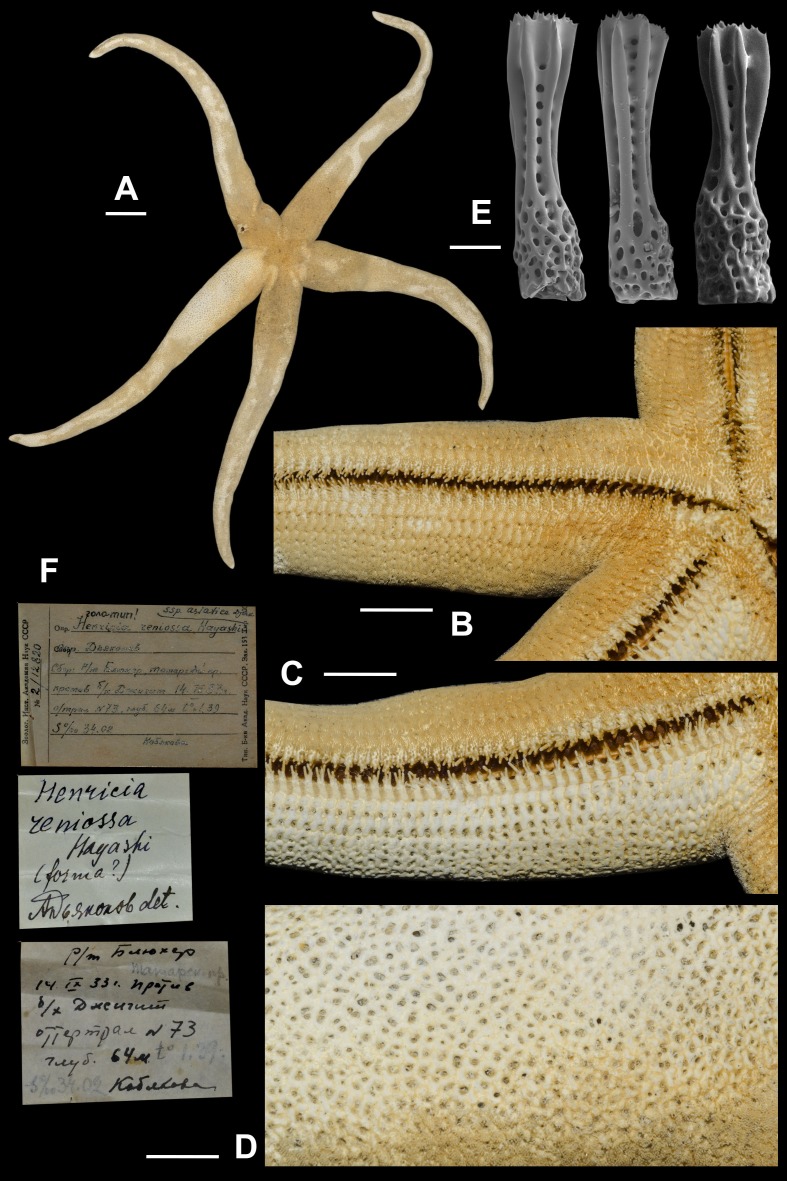
*Henricia asiatica*, lectotype. (A) abactinal view; (B) actinal side; (C) denuded actinal side; (D) abactinal side; (E) abactinal spines; (F) original labels. Scale bars: A - 20 mm, B–D - 10 mm, E - 50 µm. Photos by A. Chichvarkhin.

**8. *Henricia asiatica* Djakonov, 1958 (Fig. 9; Supplemental Information 1, first sea star: 1–5th s)**

*Henricia reniossa asiatica* Djakonov, 1958: 303–305, fig. 14; 1961: 22, pl. 2, fig. 11, pl. 12, figs. 52–53.

*Henricia asiatica* –Chichvarkhin & Chichvarkhina, 2017a: 208, Fig. 4B; Chichvarkhin & Chichvarkhina, 2017b: 25.

Type locality: Dzhygit Bay, Tatar Str., Sea of Japan, Russia.

We found a specimen (Fig. 9) in ZIN collection identified as *H. reniossa asiatica* and labeled as the “holotype”. We are designating this specimen as the lectotype of *Henricia reniossa asiatica* (Djakonov, 1958). *R* = 142 mm, *r* = 16 mm; *ZIN*–*2/12820*, Opposite Dzhygit Bay, Tatar Strait, 14 Sep 1933, 54 m depth, leg. Kobyakova.

Examined material: lectotype, *ZIN*–*2/12820*, Opposite Dzhygit Bay, Tatar Strait, 14 Sep 1933, 54 m depth, leg. Kobyakova; 1 spm, *ZIN*–7/14192, Kurile Islands, KSE, 22 Sep 1949, Shikotan Is., opposite Anama Bay, 50 m depth; 1 spm, *ZIN*–8/15941, Sea of Japan, near Kekurnyi Cape [Olga Bay], st. 39/451, 27 Oct 1931, 75–83 m depth, gravel, leg. Tarasov; 1 spm, *ZIN*, Toporok st. 93, 1949, E off Shikotan Is., 286 m depth leg. Kobyakova & Savelieva; 1 spm, *ZIN*, Dzhygit Bay, Tatar Str., o/tr [Otter-trawl] 73, 14 Sep 1933, leg. Kobyakova.

The specimen of this large sea star, whichx superficially resembles *H. (S.) asiatica* (= *H. reniossa asiatica*), had been recorded with the use of remotely controlled submersible apparatus south-east off the outer part of Vostok Bay (opposite Passek Cape, 42°44.6′N 132°43.8′E) at the depth of 45 m (appendix 1). R of the largest individual is about 90–100 mm, aboral coloration dark orange/mahogany. One of these specimens was associated with with a large mussel, *Crenomytilus grayanus* (Dunker, 1853), probably filtering plankton from water currents generated by the mollusk.

Brun (1976) reported observations interpreted as suspension feeding in *Henricia* species, hypothesizing that some *Henricia* species may engage in rheophilic feeding behavior, i.e., collecting suspended phytoplankton in water currents generated by the sponges. If further supported, this could explain why *Henricia* individuals are often encountered sitting on the sponges, which has often led to an assumed sponge diet. However, most sponges feed on microbial cells, so it remains to be demonstrated that any *Henricia* could capture such tiny food items. However, we never observed suspension feeding behavior of any *Henricia* as Rasmunsen (1965) reported for *H. sanguinolenta* (O.F. (Müller, 1776). Suspension feeding on phytoplankton is a possible feeding strategy at shallow water habitats but is poorly effective at the depths >100 m, hence this species is likely polyphagous even if they feed on phytoplankton at shallower depths.

External anatomy. large in size, R (long radius: mouth to ray tip) of lectotype 142 mm, r (short radius: mouth to interradius) 16 mm, R:r 8.9; disc small, rays long, slender, subcylindrical, tapering to sharp tips (Fig. 9A). Abactinal plates with no tubercles relatively small forming tight reticulation; plates very close set, appearing almost fused; plates crowned with about 10 blunt spines, each tipped with 5–10 very small thorns (Fig. 9E); papular areas rather small, with 1 papula (Fig. 9D); madreporite small, circular, located about 2/3 way between anus and edge of disc. Superomarginals 1/3 larger than abactinal plates bear 40–45 spines, inferomarginals heigh about twice length; twice as large as superomarginal plates and bearing 45 spines; double series of intermarginals extends about 1/3 of R, each plate bearing 15–25 spines; ventrolateral series extending full length of R. Adambulacrals (Figs. 3D, 3E) each with a single deep furrow spine and 30–35 actinal spines, 5 longer spines at furrow edge, and 10–20 smaller distally grading spines behind (Figs. 9B, 9C). Oral plates with 5 marginal and 4–5 suboral spines; in addition, there are 4–5 thick, blunt spines near the distal edge of plate. Color in life unknown, probably orange.

Distribution. It was reported by Djakonov (1961) from southern Kurile Islands and from the northwestern Sea of Japan including type locality. He also reported this species from Peter the Great Bay because of a confusion of Dzhygit Bay (Tatar Strait, actual type location) *vs*. Noviy Dzhygit Bay (Russkiy Island, Peter the Great Bay).

**Figure 10:**
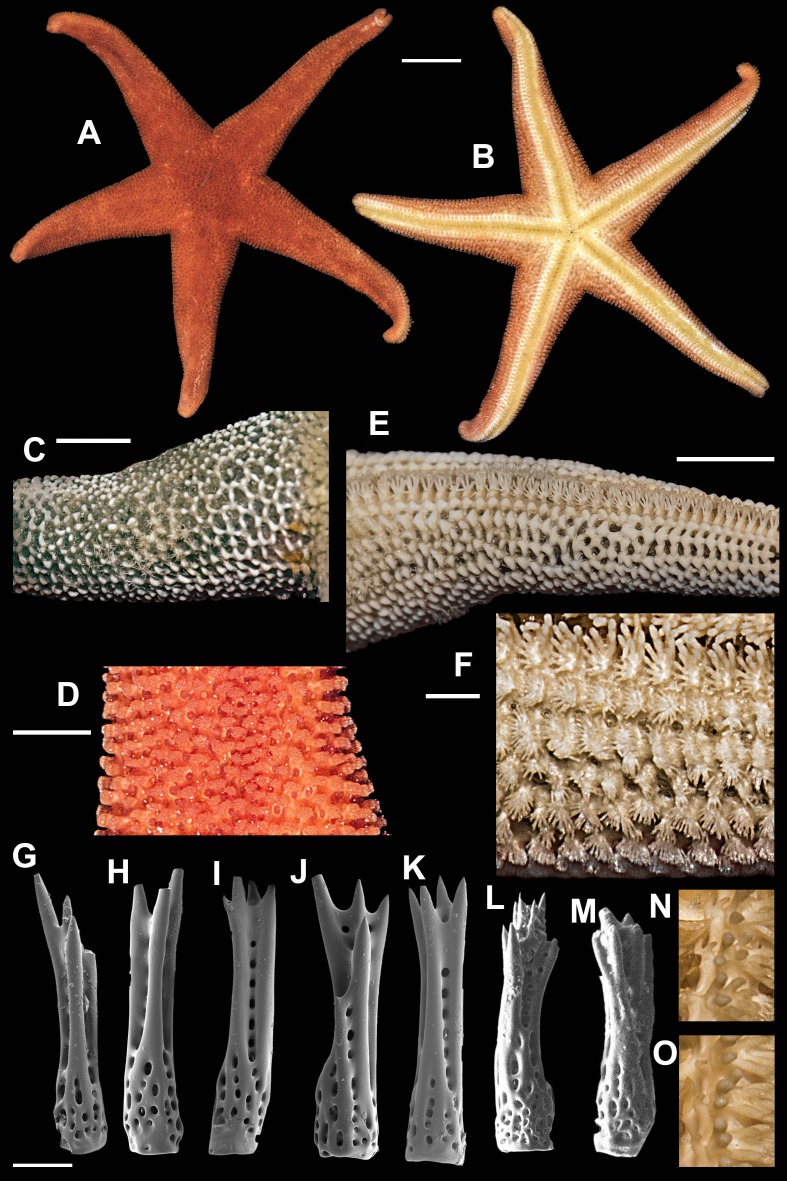
*Henricia densispina,* Vostok Bay. (A, B) life coloration; (C) denuded abactinal skeleton; (D) abactinal side of live specimen; (E) denuded actinal side; (F) actinal side of ethanol-preserved specimen; (G–K) abactinal spines; (L–M) abactinal spines of *ZIN –61/15229 H.* sp., Tatar strait, Sovetskaya Gavan; (N) distal fragment of ambulacral furrow with double deep ambulacral spines; (O) proximal fragment of ambulacral furrow with single deep ambulacral spine. Scale bars: A, B - 10 mm, C, E - 5 mm, D - 3 mm, F - 1 mm, G–M - 50 µm. Photos by A. Chichvarkhin.

**9. *Henricia (S.) densispina* (Sladen, 1878) (Fig. 10)**

**Table:** 

*Cribrella densispina* Sladen, 1878: 432, Pl. 8, figs. 5–9.
*Henricia densispina*–Fisher, 1919: 436–437, Pl. 118, figs. 2, 3, Pl. 119, figs. 1–3 (part.?); Hayashi, 1940: 140, Pl. 10, fig. 7, 8; Djakonov, 1961: 20–21, Pl.11, figs. 48–49; Xiao, Liao & Liu, 2011: 5–7, figs. 5A–5F, 12D; non Djakonov, 1958: 310, fig. 20; non Yavnov, 2010: 111–112; Chichvarkhin & Chichvarkhina, 2017b: 27, fig. I.

Type locality: Korean Strait, West coast of Japan.

Examined material: 1 spm, *MIMB–33249*, Vostok Bay, 20 Jul 2016, leg. A. Goloseyev; 1 spm; *Henricia spiculifera* as identified by Djakonov: 1 spm, *ZIN*–*61/15229*, Lesseis(?)-Data (Datta) Cape, mouth of Tumin Riv. near Imperatorskaya Gavan [Sovetskaya Gavan], 28 May 1909, leg. V. K. Arseniev; *Henricia spiculifera* det. Djakonov*:* 1 spm, *ZIN*–*1/15205*, Akademii Bay, Okhotsk Sea, 38 m, 17 Aug 1917; 3 spms, *ZIN*–*1/15155*, Okhotsk Sea, 50 m, 13 Aug 1918, leg. Meder; 3 spm, *MIMB–* 34800-34802, Vostok Bay, 06–26 Jul 2017, leg. K. Dudka; *H. tacita* det. Z.I. Baranova, ex *H. leviuscula dyscrita* det. A.M. Djakonov: 1 spm, *ZIN–20/8457 (ex 8/8457),* GGI, 3 Jun 1932, tr. 1, #14, Okhotsk Sea, 118-60 m depth, leg. Polutov; 1 spm, *ZIN–23/8455,* GGI, s/s Ara, st. 25/1, 4 Sep 1939, Okhotsk Sea, leg. Schmidt; 1 spm, *ZIN–6/8454*, GGI, s/s Ara, st. 25/8, 6 Sep 1939, Okhotsk Sea, 56°24′N 142°12′E, 80 m depth, leg. Schmidt; *H. tacita* lectotype: 1 spm, *ZIN–14/12800,* KSE st. 57, Mordvinova Bay, 2 Sep 1947, 52 m depth, on clay, sand, gravel.

External anatomy. The specimens, which we found in Vostok Bay possesses *R* = 32.5–55 mm, *r* = 7–11 mm, R: *r* = 4.6–5. Rays five, broad at base, tapering. Disc broad. Aboral skeleton widely meshed. Abactinal plates tri-lobed with well discernible tubercles. Abactinal pseudopaxillae with 18–25 spines in 3–5 rows. Papular areas with 1–2 papulae. Abactinal spines slender, 450 µm long, 80–90 µm wide, with 3–4 long fine apical thorns (Figs. 10G– 10K), not covered by skin membrane.

Marginal rows irregular proximally, after a half of ray length well discernible. Superomarginal row, consists of plates similar but slightly larger than adjacent aborals, bearing about 25 spines, same as abactinal spines, arranged in four irregular rows. Intermarginal row extended to full ray length; plates with about 20 spines in one row. Plates of intermarginal row oval, smaller than superomarginals. Inferomarginal plates relatively large, transversally elongated, with ridges. pseudopaxillae bear 30–35 spines in 4–5 irregular rows.

Ventrolateral plates round or oval; pseudopaxillae bear about 20 spines in four irregular rows. Adambulacrals with three larger near-furrow spines and 8–9 smaller spines in three rows (Fig. 9F). Deep furrow spine small, single but double at ray tip (Figs. 10N, 10O). Oral plates each bear single apical, four marginal, and three suboral spines.

Color in life. Abactinal and actinal sides bright orange (Figs. 10A, 10B). Anal area darker than background; madreporite light creamy. Light yellow to orange at near-furrow area.

Ecology. The species was found on rocky substrate at the depth of 10 m at water temperature 20 °C.

Distribution. Known from the northwestern part of the Sea of Japan and South China Sea (Xiao, Liao & Liu, 2011) and Japan (Hayashi, 1940). Similar species previously reported by Djakonov (1961) from Tatar Strait as *H. spiculifera* and *H. densispina* likely belongs to a different species possessing short and more stout spines with usually four apical thorns. A species inhabiting the Sea of Japan referred to as *H. spiculifera* or *H. leviuscula spiculifera* (Hayashi, 1940; Djakonov, 1961) and probably from South China Sea (Xiao, Liao & Liu, 2011) belong to a distinct species possessing very fine slender spines crowned with 3 long thorns, while in *H. densispina* the spines are stouter (Xiao, Liao & Liu, 2011).

The specimens from Vostok Bay also resemble *H. leviuscula dyscrita* (Fisher, 1911) described from California. This name was reported by Djakonov (1948) from the western Pacific, Okhotsk Sea. Later, he did not mention this name in his publications. We found three specimens in ZIN collection identified as *H. leviuscula dyscrita,* but later they were re-identified as *H. tacita* Djakonov, 1958. We compared these specimens with the specimen *ZIN–14/12800* marked as “*H. tacita* holotype” presumably by Z.I. Baranova after A.M. Djakonov’s death (although the holotype was not designated for this name by the author): this specimen from Aniva Bay, off Sakhalin Island possess less discernible superomarginal plates, the ridges on proximal marginal plates, and wider rays than re-identified exemplars from Okhotsk Sea, i.e., this specimen strikingly resembles *H. reniossa* Hayashi, 1940 described from northern Japan, although these two species possess very distinctive abactinal spines. There are four extra “*H. leviuscula dyscrita* det. Djakonov*”* specimens from Okhotsk are listed in ZIN catalogue (*ZIN–4/3453* and *ZIN–10/8450)* collected by Ushakov in 1932, but we did not succeed in finding them. All these specimens are likely the syntypes of *H. tacita* Djakonov, 1958. To avoid future confusion, we are designating the lectotype for *H. tacita* Djakonov, 1958 with the specimen *ZIN–14/12800* because it fully conforms to Djakonov’s original diagnosis and photographed specimen (Djakonov, 1961: Pl. 11, fig. 44).

**Table 2:** Number and distribution of recorded specimens in this study.

Species	Inner part of the bay (2015)	Outer part of the bay (2015/2016/2017)	Bottom
*alexeyi*	0	2∕1∕2	nearshore boulders
*pacifica*	0	5∕0∕0	rocks, pinnacles
*nipponica*	8	29∕15∕1	various rocky
*granulifera*	0	1∕0∕1	rocks, pinnacles
*asiatica*	0	1? /0 /0	silty
*djakonovi*	52	324∕21∕8	various rocky
*hayashii*	1	14∕2∕0	rocks, pinnacles
*densispina*	0	0∕1∕3	rocks, pinnacles

The specimens from Vostok Bay are different from all specimens mentioned above. They do not possess crescent-shaped pseudopaxillae and smaller papular areas, i.e., do not belong to *H. tacita* or *H. leviuscula dyscrita*.

### Abundance and spatial distribution of *Henricia* in Vostok Bay

During 2010, 2014–2017, we recorded 537 individuals of *Henricia* spp. in Vostok Bay (Table 2). Two of these individuals of *Henricia* sp. were recorded by a submersible apparatus, although trawling in this area did not allow us to collect any *Henricia* species. Just three species were found in periodically desalted (32‰ to 2‰) inner part of the bay, while the others were strictly associated with always highly mineralized (appr. 32‰) outer part, and occurred on mainly rocky bottoms but sometimes on a silty substrate.

#### Key to the genus *Henricia* of Vostok Bay

**Table:** 

1a Spines not covered by thick skin membrane; spines in abactinal and inferomarginal pseudopaxillae in 3 or more rows or in small pseudopaxillae with 3–5 spines, spines with no lateral thorns ................................... **subgenus*Setihenricia***Chichvarkhin & Chichvarkhina, 2017a; Chichvarkhin & Chichvarkhina, 2017b (2)
1b Abactinal spines covered by thick skin membrane, spines in double or single rows along plate ridges, spines with lateral thorns ............................................. 5
2a At least a half of abactinal pseudopaxillae are crescent-shaped (reniform).......................................3
2b The pseudopaxillae are not reniform. Abactinal pseudopaxillae circular, closely set leaving space for one or two papulae, with 10–15 long spines crowned with 3–4 sharp thorns; disc broad; rays tapering; color bright orange to pink .............................................. ***H. (S.) densispina*** (Sladen, 1878)
3a Inferomarginal plates lack ridges or tubercles, color orange to brown with wide almost back stripes. .................................................................... ***H. (S.) djakonovi***Chichvarkhin & Chichvarkhina, 2017a; Chichvarkhin & Chichvarkhina, 2017b 3
3b Inferomarginal plates with transversal ridges, well developed proximally, color bright orange .............................4
4a Abactinal spines with a crown of relatively large thorns, R <60 mm .................................................................................. ***H. (S.) hayashii***Djakonov, 1961
4b Abactinal spines large with blunt tip with tiny thorns, R may exceed 100 mm ...................................................................... ***H. (S.) asiatica***Djakonov, 1958
5a Marginal rows bent, poorly discernible or rudimental; abactinal and inferomarginal spines in pseudopaxillae with 3–5 spines; spines with lateral thorns; color brown, spine apices, oral, adambulacral and ventrolateral areas bright orange .......................................................................................... (***H. Aleutihenricia) granulifera***Hayashi, 1940
5b Marginal rows well discernible ......................... **subgenus*Henricia***Gray, 1840 (7)
6a Inferomarginal plates transversally elongated, 3–4-fold larger then ventrolaterals; Abactinal spines in one row; 2–3 papulae in an area; intermarginal row extends to full ray length; additional plates between intermarginal, inferomarginal, and superomarginal rows present; papulae very long; color brown, dirty orange .................................... ***H. (H.) nipponica***Uchida, 1928.
6b Inferomarginal plates not larger then ventrolaterals, transversally not elongated ...........................................................7
7a Abactinal spines in one row; superomarginal plates similar to abactinal; color bloody red, proximally, on ray tips, and on disc may be with white tint. Abactinal spines covered by very thick skin; mainly one papula in an area; ................................ ***H. (H.) pacifica***Hayashi, 1940
7b Abactinal spines in two rows, some pseudopaxillae with one row .............................8
8a Rays thick; superomarginal plates small, similar to adjacent plates. One or few intermarginal rows extended to less than half of ray length. Wide-meshed abactinal skeleton with 2–3 papulae in an area. Coloration varies greatly: yellow-orange, pink, red, also in combinations of these colors ................................................................................. ***H. (H.) oculatarobusta***Djakonov, 1958
8b Rays slim; proximal adambulacral, ventrolateral and inferomarginal spines joined in crests by skin membrane; tightly-meshed abs; coloration variess: yellow and orange on disk and tips or red and white on disk or uniformly orange, ventral side lighter colored ..................................................................................... ***H. (H.) alexeyi***Chichvarkhin & Chichvarkhina, 2017a; Chichvarkhin & Chichvarkhina, 2017b

### Discussion

Smirnov’s (2013) review of the invertebrates of Russian Pacific waters estimates that there are 21 echinasterid species reported from the Sea of Japan. Some of them were confirmed to the eastern part of that sea (*H. aniva* Djakonov, 1958, *H. pseudoleviuscula* Djakonov, 1958, *H. sachalinica* Djakonov, 1958, *H. tacita* Djakonov, 1958, *H. orientalis* Djakonov, 1950). Some of the listed species are dubious reports, including some that were likely misidentified (*H. spiculifera* Clark, 1901, *H. aspera robusta* Djakonov, 1948). Occurrence of deep-water *H. reniossa asiatica* Djakonov, 1961, which very likely is a distinct species related to a shallow-water *H. reniossa* Hayashi, 1940, is possible in Peter the Great Bay but we did not find it in Vostok Bay, probably because we focused mostly on shallow depths. Therefore, the total number of species that we have confirmed for Vostok Bay or its vicinity is estimated to be less than or about 15 species. Several *Henricia* species are known from adjacent waters of Korea and Japan, e.g*. H. ohshimai* Hayashi, 1940, *H. reniossa* Hayashi, 1940*, H. nipponica* Uchida, 1928, so they could also be found here.

In general, *Henricia* sea stars are common in Vostok Bay. However, *H. djakonovi* dominates among the others making about 80% of found individuals (Chichvarkhin, 2017a). The second abundant species is *H. nipponica* (about 15% of found individuals), while the others are rather rare (Table 2). The *H. densispina, H. hayashii,* and *H. granulifera* were encountered within this study just once despite the fact that the two latter species were found abundant in Rudnaya Bay, where water salinity is high and remains stable throughout the year. We hypothesize that most *Henricia* species are intolerant of decreased salinity in the inner part of Vostok Bay, and are restricted to the outer bay where the salinity was closer to oceanic. All species treated here inhabit near-shore rocky areas, with none found on the shallow silty bottom that completely occupies the central part of Vostok Bay and its inner part. The only exceptions were two remotely-recorded exemplars from deeper water on a silty bottom.

### Conclusions

This study revealed seven *Henricia* species that inhabit Vostok Bay, with two additional species suspected as present but not yet sampled. All these species can be clearly identified with DNA barcoding approach using 16S rRNA marker. Morphological characters are also very valuable for identification of most species. Vostok Bay does not constitute a good habitat for high *Henricia* species diversity because of relatively low salinity water, especially in summer time, less than 30 m depths, and silty bottoms. However, a discovery of a few overlooked species remains possible along the outer shore of the bay.

##  Supplemental Information

10.7717/peerj.6585/supp-1 Supplemental Information 1 Supplement 1 *Henricia* cf. *asiatica* recorded in the outer part of Vostok Bay. Footage by Nikolay Kashenko.

10.7717/peerj.6585/supp-2 Supplemental Information 2 Full 16S dataset in Fasta format

## Additional Information and Declarations

### Data Availability

The following information was supplied regarding data availability: GenBank: KY464037–KY464043, MG976220–MG976226, and KY744469–KY74446. The full 16S dataset in FASTTA format is provided as a Supplemental File.

## References

[ref-1] Adrianov AV, Kussakin OG (1998). A check-list of biota of the Peter the Great Bay, the Sea of Japan.

[ref-2] Brun E (1976). Ecology and taxonomic position of *Henricia oculata* Pennant. Thalassia Jugosl.

[ref-3] Chichvarkhin A (2017a). *Henricia djakonovi* sp. nov. (Echinodermata, Echinasteridae): a new sea star species from the Sea of Japan. PeerJ.

[ref-4] Chichvarkhin A (2017b). Sea star *Henricia spiculifera* (Clark, 1901) in the northwestern Pacific: one species or three?. PeerJ.

[ref-5] Chichvarkhin A, Chichvarkhina O, Dautova TN (2017a). A new sea star species of the genus *Henricia* Gray, 1840 (Echinodermata, Asteroidea) from the northwestern Sea of Japan and description of a new subgenus. Life-supporting Asia-Pacific marine ecosystems, biodiversity and their functioning.

[ref-6] Chichvarkhin A, Chichvarkhina O (2017b). Sea stars of the genus *Henricia* Gray, 1840 (Echinodermata, Asteroidea) from the northwestern Sea of Japan.

[ref-7] Chichvarkhin A, Chichvarkhina O, Ekimova I, Chalenko K (2016). First record of nudibranch mollusk *Onchidoris muricata* (OF Müller, 1776) (Mollusca, Gastropoda, Heterobranchia) in the Sea of Japan and its ephemeral population associated with unusual prey. Marine Biodiversity.

[ref-8] Clark AH (1916). Six new starfishes from the Gulf of California and adjacent waters. Proceedings of Biological Society of Washington.

[ref-9] Clark HL (1901). Echinoderms from Puget Sound, observations made on the echinoderms collected by the parties from Columbia University, in Puget Sound in 1896 and 1897. Proceedings of Boston Society of Natural History.

[ref-10] Clark RN, Jewett SC (2010). A new genus and thirteen new species of sea stars (Asteroidea: Echinasteridae) from the Aleutian Island Archipelago. Zootaxa.

[ref-11] Dautov SS, Tyurin SA (2014). The echinoderms of Vostok Bay in the Sea of Japan. Biota i Sreda Zapovednikov Dalnego Vostoka.

[ref-12] Djakonov AM (1948). Key to the echinoderms of the Far Eastern Seas. Izvestiya TINRO.

[ref-13] Djakonov AM (1950). Sea stars of the seas of the USSR. Keys to the Fauna of the USSR.

[ref-14] Djakonov AM (1958). Echinoderms (Echinodermata), except holothurians, collected by Kuril-Sakhalin expedition in 1947–1949. Issledovaniya Delnevostochnykh Morei SSSR.

[ref-15] Djakonov AM (1961). Review of sea stars of the genus *Henricia* Gray from the northwestern parts of Pacific Ocean. Issledovaniya Delnevostochnykh morei SSSR.

[ref-16] Ekimova I, Valdés Á, Schepetov D, Chichvarkhin A (2016). Was Gordon Robilliard right? Integrative systematics suggests that *Dendronotus diversicolor* Robilliard, 1970 is a valid species. Canadian Journal of Zoology.

[ref-17] Fedorov SN, Shubina LK, Kicha AA, Ivanchina NV, Stonik VA, Kwak JI, Jin JO, Bode AM, Dong Z (2008). Proapoptotic and anticarcinogenic activities of leviusculoside g from the starfish *Henricia leviuscula* and probable molecular mechanism. Natural Products Communications.

[ref-18] Fisher WK (1911). Asteroidea of the North Pacific and adjacent waters. Part 1. Phanerozonia and Spinulosa. Bulletin of the United States National Museum.

[ref-19] Fisher WK (1919). Starfishes of the Philippines seas and adjacent waters. Bulletin of the United States National Museum.

[ref-20] Fisher WK (1928). Asteroidea of the North Pacific and adjacent waters. Part 2. Forcipulata (Part). Bulletin of the United States National Museum.

[ref-21] Fisher WK (1930). Asteroidea of the North Pacific and adjacent waters. Part 3. Forcipulata (Concluded). Bulletin of the United States National Museum.

[ref-22] Folmer O, Black M, Hoeh W, Lutz R, Vrijenhoek R (1994). DNA primers for amplification of mitochondrial cytochrome c oxidase subunit I from diverse metazoan invertebrates. Molecular Marine Biology and Biotechnology.

[ref-23] Gray JE (1840). A synopsis of the genera and species of the Class Hypostoma (Asterias Linn). Annual Magazine of Natural History, Series 1.

[ref-24] Hayashi R (1940). Contributions to the classification of the sea-stars of Japan. I. Spinulosa. Journal of the Faculty of Science, Hokkaido Imperial University, Series VI: Zoology.

[ref-25] Hayashi R (1952). Sea stars of Seto and adjacent waters. Publications of the Seto Marine Biological Laboratory.

[ref-26] Heding SG (1935). Echinoderms. The scoresby Sound committee’s 2nd East Greenland expedition in 1932 to King Christian IX’s Land. Meddelelser om Grønland.

[ref-27] Jewett SC, Clark RN, Chenelot E, Harper S, Hoberg MK (2015). Field guide to sea stars of the Aleutian Islands.

[ref-28] Knott KE, Ringvold H, Blicher ME (2017). Morphological and molecular analysis of Henricia Gray, 1840 (Asteroidea: Echinodermata) from the Northern Atlantic Ocean. Zoological Journal of the Linnean Society.

[ref-29] Koehler R (1907). Note preliminaire sur quelques Asteries et Ophiures provenantdes campagnes de la Princesse Alice. Bulletin de l’Institut océanographique de Monaco.

[ref-30] Kumar S, Stecher G, Tamura K (2016). MEGA7: molecular evolutionary genetics analysis version 7.0. Molecular Biology and Evolution.

[ref-31] Laakmann S, Boos K, Knebelsberger T, Raupach MJ, Neumann H (2016). Species identification of echinoderms from the North Sea by combining morphology and molecular data. Helgoland Marine Research.

[ref-32] Larkin MA, Blackshields G, Brown NP, Chenna R, McGettigan PA, McWilliam H, Valentin F, Wallace IM, Wilm A, Lopez R, Thopson JD, Gibson TJ, Higgins DG (2007). Clustal W and Clustal X version 2.0. Bioinformatics.

[ref-33] Layton KKS, Corstorphine EA, Hebert PDN (2016). Exploring Canadian echinoderm diversity through DNA barcodes. PLOS ONE.

[ref-34] Lebedev EB (2015). The echinoderms (Invertebrata, Echinodermata) of the Far Eastern Marine State Reserve. Biota i Sreda Zapovednikov Dalnego Vostoka.

[ref-35] Madsen FJ (1987). The *Henricia sanguinolenta* complex (Echinodermata, Asteroidea) of the Norwegian Sea and adjacent waters. A re-evaluation, with notes on related species. Steenstrupia.

[ref-36] Müller OF (1776). Zoologiae Danicae prodromus seu Animalum Daniae et Norvegiae indigenarum characteres, nomina, et synonyma imprimis popularium.

[ref-37] Palumbi S (1996). Nucleic acids II: the polymerase chain reaction. Molecular systematics, 2nd edition.

[ref-38] Perrier E (1884). Mémoire sur les étoiles de mer recueilliés dans la mer des Antilles et le golfe du Mexique: durant les expéditions de dragace faites sous la direction de M. Alexandre Agassiz. Archives (Muséum national d’histoire naturelle (France)), 2.

[ref-39] Rasmunsen BN (1965). On Taxonomy and Biology of the North Atlantic Species of the 399 Asteroid Genus *Henricia* Gray. Meddelelser fra Danmarks Fiskeri- og Havundersøgelser. Ny 400 serie.

[ref-40] Shin S, Rho BJ (1996). Illustrated encyclopedia on fauna and flora of Korea. 36 Echinodermata.

[ref-41] Shin S, Ubagan MD (2015a). A newly recorded sea star of genus *Henricia* (Asteroidea: Spinulosida: Echinasteridea) from the East Sea of Korea. Korean Journal of Environmental Biology.

[ref-42] Shin S, Ubagan MD (2015b). A newly recorded sea star of genus *Henricia* (Asteroidea: Spinulosida: Echinasteridae) from Jeju Island, Korea. Korean Journal of Environmental Biology.

[ref-43] Sladen WP (1878). On the Asteroidea and Echinoidea of the Korean Seas. Journal of the Linnean Society (Zoology).

[ref-44] Smirnov AV (2013). Class Asteroidea, Check list of free-living invertebrates of Russia. Issledovaniya Fauny Morei.

[ref-45] Stimpson W (1857). On the crustacea and echinodermata of the pacific shores of North America. Boston Jouurnal of Natural History.

[ref-46] Temereva E, Chichvarkhin A (2017). A new phoronid species, *Phoronis embryolabi*, with a novel type of development, and consideration of phoronid taxonomy and DNA barcoding. Invertebrate Systematics.

[ref-47] Ubagan MD, Shin S (2016). A new record of sea star genus Hen-ricia (Asteroidea: Spinulosida: Echinasteridae) from Jeju Island, Korea. Journal of Species Research.

[ref-48] Uchida T (1928). Report of the biological survey of Mutsu Bay. 11. Starfishes of Mutsu Bay. Scientific Reports of Tohoku Imperial University, Ser. 4.

[ref-49] Utkina NK (2009). Anthraquinoid pigments from the deep-water starfish *Henricia* sp. Chemistry of Natural Compounds.

[ref-50] Verrill AE (1870). Notes on Radiata in the Museum of Yale College. 1. Descriptions of new starfishes from New Zealand. Transactions of the Connecticut Academy of Arts and Sciences.

[ref-51] Verrill AE (1909). Remarkable development of starfishes on the northwest American Coast: hybridism; multiplicity of rays; teratology; problems in evolution; geographical distribution. American Naturalist.

[ref-52] Verrill AE (1914). Monograph of the shallow-water starfishes of the North Pacific coast from the Arctic Ocean to California. Harriman Alaska Series.

[ref-53] Xiao N, Liao Y, Liu R (2011). Records of the genus *Henricia* Gray, 1840 (Echinodermata: Asteroidea: Echinasteridae) from Chinese waters. Zootaxa.

[ref-54] Yavnov SV (2010). Atlas of the sea stars of the Far Eastern seas of Russia.

